# Elevated FBXL6 expression in hepatocytes activates VRK2-transketolase-ROS-mTOR-mediated immune evasion and liver cancer metastasis in mice

**DOI:** 10.1038/s12276-023-01060-7

**Published:** 2023-09-01

**Authors:** Jie Zhang, Xiao-Tong Lin, Hong-Qiang Yu, Lei Fang, Di Wu, Yuan-Deng Luo, Yu-Jun Zhang, Chuan-Ming Xie

**Affiliations:** Key Laboratory of Hepatobiliary and Pancreatic Surgery, Institute of Hepatobiliary Surgery, Southwest Hospital, Third Military Medical University (Army Medical University), Chongqing, 400038 China

**Keywords:** Liver cancer, Ubiquitylation, Phosphorylation, Ubiquitylation, Protein-protein interaction networks

## Abstract

Metastatic hepatocellular carcinoma (HCC) is the most lethal malignancy and lacks effective treatment. FBXL6 is overexpressed in human hepatocellular carcinoma (HCC), but whether this change drives liver tumorigenesis and lung metastasis in vivo remains unknown. In this study, we aimed to identify FBXL6 (F-Box and Leucine Rich Repeat Protein 6) as a key driver of HCC metastasis and to provide a new paradigm for HCC therapy. We found that elevated FBXL6 expression in hepatocytes drove HCC lung metastasis and was a much stronger driver than Kras mutation (*Kras*^*G12D/+*^*;Alb-Cre)*, p53 haploinsufficiency (*p53*^*+/-*^) or Tsc1 loss (*Tsc1*^*fl/fl*^*;Alb-Cre*). Mechanistically, VRK2 promoted Thr287 phosphorylation of TKT and then recruited FBXL6 to promote TKT ubiquitination and activation. Activated TKT further increased PD-L1 and VRK2 expression via the ROS-mTOR axis, leading to immune evasion and HCC metastasis. Targeting or knockdown of TKT significantly blocked FBXL6-driven immune evasion and HCC metastasis in vitro and in vivo. Notably, the level of active TKT (p-Thr287 TKT) was increased and was positively correlated with the FBXL6 and VRK2 expression levels in HCC patients. Our work provides novel mechanistic insights into FBXL6-driven HCC metastasis and suggests that targeting the TKT-ROS-mTOR-PD-L1/VRK2 axis is a new paradigm for treating patients with metastatic HCC with high FBXL6 expression.

## Liver cancer: Clean-up protein drives growth and spread

The activity of FBXL6, a protein involved in cellular clean up mechanisms, is a significant driver of liver cancer growth and spread. Researchers led by Chuan-Ming Xie from the Southwest Hospital of Army Medical University in Chongqing, China, demonstrated that increased levels of FBXL6 are linked to unfavorable outcomes in liver cancer patients and contribute to the spread of liver tumors in mice. FBXL6 achieves these effects by attaching small tags to the enzyme transketolase (TKT), thereby activating it. Consequently, TKT sets off a series of molecular events that promote cancer spread and suppress the immune recognition of tumor cells. Encouragingly, a drug capable of inhibiting TKT effectively blocked FBXL6-driven spread in both human cell cultures and mouse models, offering a promising therapeutic approach for treating advanced liver cancer.

## Introduction

Hepatocellular carcinoma (HCC) is a major cause of death worldwide^1^. The five-year survival rate of HCC is less than 10%^2^. The lack of effective treatment is a main reason for the high mortality of HCC. Currently, there are only two types of FDA-approved drugs for advanced HCC, namely, multikinase inhibitors and immune checkpoint inhibitors. However, oral multikinase inhibitors (sorafenib, regorafenib and lenvatinib) can increase the median overall survival (OS) time for less than four months in 10% of HCC patients^3,4^. PD-1/PD-L1-targeted immune checkpoint inhibitors (nivolumab and pembrolizumab) have beneficial effects in only 30% of HCC patients^5^. Therefore, exploring the mechanisms underlying the initiation and development of HCC and identifying effective target genes are particularly important for the prevention and treatment of HCC.

Ubiquitination plays a critical role in many physiological processes, including cell survival, cell differentiation, and metabolic homeostasis, by modulating protein stability and subcellular localization^6,7^. Abnormal regulation of ubiquitination causes a series of diseases and conditions, including cancer, NASH, and obesity^8^. E3 ubiquitin ligases (E3s) have attracted increasing attention as potential therapeutic targets in cancer^9,10^. However, except for several well-defined E3s, e.g., FBXW7^11,12^ and SKP2^13^, most E3s remain poorly characterized; thus, fully understanding the ubiquitination system in cancers is highly challenging. A recent study reported that the E3- FBXL6 (F-Box and Leucine Rich Repeat Protein 6) was highly expressed in liver cancer and promoted the proliferation of SMMC7721 and Hep3B hepatocellular cancer cells in vitro^14^. However, it whether FBXL6 spontaneously promotes the development of liver tumors and metastasis in vivo and the mechanism underlying this action remain unknown. Here, we report that FBXL6 drives HCC metastasis in vivo and that its effect is stronger than that of mutant Kras^G12D^. We believe that gaining an understanding the role of FBXL6 in hepatocarcinogenesis will be beneficial for patients with metastatic HCC.

Transketolase (TKT) is a ubiquitous enzyme that catalyzes the reversible transfer of two-carbon ketol units between ketose and aldose phosphates^15^. It controls carbon flux through the nonoxidative branch of the pentose phosphate pathway (PPP) and provides raw materials for DNA and RNA synthesis in tumor cells^16,17^. The human genome contains three genes encoding TKT isozymes: TKT, TKTL1 and TKTL2^18^. TKT is expressed not only in normal organs but also in most tumor tissues, including HCC tissues, in humans, whereas the other two isozymes (TKTL1 and TKTL2) are expressed mainly in the testis^19^. Evidence has shown that TKT is overexpressed in various cancers, including breast cancer^20^, cervical cancer^21^, and liver cancer^22^. High TKT levels were found to be associated with poor survival^22^. Furthermore, inhibition or knockdown of TKT slowed the growth of tumors^2^. However, to our knowledge, whether the oncogenic activity of TKT depends on its ubiquitination remains unknown.

VRK2 (vaccinia-related kinase 2) is a novel Ser-Thr kinase^23^. VRK2 can interact with phosphorylated AKT after autophagy induction, controlling cellular proliferation and mitochondrial outer membrane stabilization^24^. Low levels of VRK2 increase cell sensitivity to apoptosis induction by chemotherapeutic drugs such as camptothecin and doxorubicin^23^. VRK2, as an active kinase, plays a role in the regulation of cancer cell invasion through the NFAT pathway and COX-2 expression^25^. VRK2 is highly expressed in some carcinomas^26,27^, but little is known about the mechanism underlying this expression pattern. Ran is a novel negative regulator of nuclear VRK kinase activity^28^. Here, we report that VRK2 phosphorylates TKT on Thr287, which enhances its enzyme activity, leading to upregulation of VRK2 expression.

Here, using an *Fbxl6* knock-in mouse model and HCC patient tissues, we demonstrate the crucial roles of FBXL6 in liver cancer development and show by example that blocking TKT would be sufficient to impede HCC development in a subset of HCC patients with high FBXL6 expression.

## Materials and methods

### Human cancer samples

Hepatocellular carcinoma tissues from patients were obtained at Southwest Hospital, Chongqing, China. Sample diagnoses were confirmed histologically. Samples were fixed with paraformaldehyde solution and embedded in paraffin for histological analysis. The study protocol conformed to the ethical guidelines of the 1975 Declaration of Helsinki as reflected by a priori approval by the local Ethics Committee (Third Military Medical University) (KY2020127). Written informed consent was obtained from each patient. Detailed clinical and pathological data for each patient were obtained.

### Animal studies on FBXL6-induced HCC metastasis and TKT inhibition

*Alb-Cre* (016833) mice with liver-specific Cre recombinase expression were obtained from The Jackson Laboratory (Bar Harbor, Maine, USA). Fbxl6 ^*LSL-fl/+*^ mice were generated by Beijing Biocytogen Co. on its CRISPR‒Cas9 gene targeting platform. To generate this mouse strain, fertilized mouse zygotes were coinjected with a mixture of Cas9 mRNA, a guide RNA (gRNA) targeting the mouse ROSA26 gene, and a construct containing a CAG promoter-LoxP-STOP-LoxP-mouse Fbxl6 cDNA cassette. Injected zygotes were transferred into pseudopregnant C57BL/6 N mice. Mice with correct gene targeting were identified by PCR and gene sequencing. Then, *Alb-cre* mice were bred with *Fbxl6*^*LSL-fl/+*^ mice to generate *Fbxl6*^*LSL-fl/+*^*;Alb-Cre* (*Fbxl6;Alb-Cre*) mice with high FBXL6 expression. Correct gene targeting in these mice was confirmed by PCR. *Fbxl6;Alb-Cre* and *Alb-Cre* mice were observed for 310 days to evaluate the role of FBXL6 in tumorigenesis and lung metastasis.

To establish the orthotopic tumor model^29^, primary liver cells were successfully isolated from *Fbxl6;Alb-Cre* mice. Subsequently, 5×10^6^ cells were suspended in 100 µl of PBS/Matrigel (1:1) and transplanted into the hypodermis of 6-week-old male nude mice (GemPharmatech, Chengdu, China). After two weeks, the nude mice were euthanized with an excessive dose of anesthetic, and the tumor tissue was removed. The tumors were cut into 1 mm^3^ pieces, which were retransplanted into the livers of anesthetized 16 six-week-old male C57BL/6 N mice by laparotomy. Three days after initiation of orthotopic tumorigenesis, treatment with the TKT inhibitor N3PT (diluted in 0.9% NaCl, 25 mg/kg i.p. every other day for 3 weeks) was begun. Finally, the mice were euthanized with an excessive dose of anesthetic. The tumors were isolated, imaged, and measured with an electronic caliper for calculation of the tumor volume. The tumors were also weighed.

To evaluate the effect of TKT knockdown on FBXL6-induced hepatocarcinogenesis, 5-week-old *Fbxl6;Alb-Cr*e mice were treated with DEN (25 mg/kg), followed by 28 injections of CCl_4_ (0.5 ml/kg) alone or in combination with adeno-associated virus AAV9-shTKT-eGFP-TBG (5×10^10^; liver-restricted expression) after 4 injections of CCl_4_. DEN/CCl_4_ treatment was used to shorten the tumorigenesis observation periods. After the mice were sacrificed, the livers were photographed and weighed to calculate the liver/body weight ratio. The tumors were counted, and the largest tumor diameter was measured with a caliper. The tumors were also weighed.

All mice were maintained in pathogen-free facilities in the Animal Room of Southwest Hospital. Animals were randomly allocated to experimental groups. The animal experiments were performed in a blinded manner. All animal experiments were approved by the Institutional Animal Care and Use Committee (IACUC) of Army Medical University and were performed in compliance with all relevant ethical regulations (AMUWEC2020022).

### Statistical analysis

The results are presented as the means ± SEMs. Comparisons between two groups were performed using two-tailed unpaired Student’s *t* test. Comparisons among more than two groups were performed using ANOVA with Tukey’s test or the Bonferroni correction for multiple comparisons. Fisher’s exact test and the χ^2^ test were used to assess the association between FBXL6 and TKT expression. Overall survival (OS) was estimated using the Kaplan‒Meier method, and survival was compared between the groups using the log-rank test with GraphPad Prism, version 8.0 (GraphPad Software, San Diego, CA). Cox proportional hazards regression models were used to calculate the hazard ratios (HRs) and 95% confidence intervals (CIs). Multivariate Cox proportional hazards regression analysis was used to identify independent prognostic factors. Data were analyzed with SPSS Viewer 24.0 (IBM, IBM, Armonk, NY). The significance threshold was set as *p* < 0.05, and significance is presented as *p < 0.05, **p < 0.01, and ***p < 0.001.

For in vivo experiments, an investigator treated the mice and collected the tissue samples. These samples were assigned code numbers. The analyses, including RT‒qPCR, H&E staining, and IHC staining, were performed by another independent investigator. All Western blotting and RT‒qPCR analyses were independently repeated two or three times.

### Further methods

A detailed methods section is provided in the supplementary materials of this manuscript.

## Results

### FBXL6 is an independent risk factor for aggressive HCC and drives HCC lung metastasis in vivo significantly more strongly than Kras mutation, p53 loss or Tsc1 loss

**Fig. 1:**
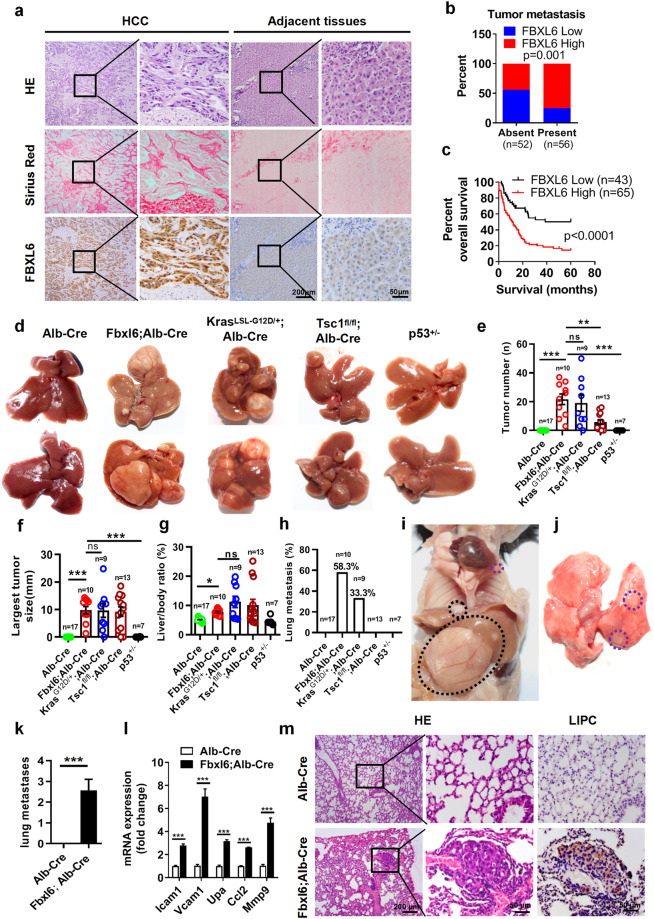
FBXL6 is an independent risk factor for aggressive HCC and drives HCC lung metastasis in vivo significantly more strongly than Kras mutation, p53 loss or Tsc1 loss. **a** Histologic analysis hematoxylin and eosin (H&E) and Sirius red staining and evaluation of FBXL6 expression by IHC staining in liver cancer tissues and adjacent normal tissues from 108 patients. Scale bars, 200 μm or 50 μm. **b** Association between FBXL6 protein expression and metastasis. **c** Association between FBXL6 protein expression and overall survival in HCC patients. The data are presented as the means ± SEMs. The log-rank (Mantel‒Cox) test was used, *p* < 0.0001. **d**–**h** *Alb-Cre* (*n* = 17), *Fbxl6;Alb-Cre* (*n* = 10), *Kras*^*LSL-G12D/+*^*;Alb-Cre* (*n* = 9), *Tsc1*^*fl/fl*^*;Alb-Cre* (*n* = 13), and *p53*^*+/*–^ (*n* = 7) female mice were monitored for 310 days and were then euthanized. The livers were imaged (**d**). The liver tumor number (**e**), largest tumor size (**f**), liver/body weight ratio (**g**), and rate of lung metastasis (**h**) were analyzed. **i**, **j** Representative images showing lung metastasis in 310-day-old *Fbxl6;Alb-Cre* mice. **k** The number of observed metastases in mice of each genotype was quantified. **l** qPCR results showing the elevated expression of the metastasis markers *Icam1, Vcam1, Upa, Ccl2*, and *Mmp9* in liver tumors of *Fbxl6;Alb-Cre* mice. *n* = 6. **m** Representative H&E and IHC staining images showing distant lung metastatic foci expressing the hepatocytic marker LIPC. Scale bars, 200 μm or 50 µm. The data are presented as the means ± SEMs. One-way ANOVA with the Bonferroni correction for multiple comparisons was used in (**e**–**g**); two-way ANOVA with the Bonferroni correction for multiple comparisons was used in (**l)**; two-tailed unpaired t test was used in (**k**). ns nonsignificant. *p ≤ 0.05, **p ≤ 0.01, ***p ≤ 0.001.

A previous study indicated that FBXL6 was highly expressed in HCC tissues, but whether FBXL6 protein expression is an independent risk factor in metastatic HCC remains unclear. Here, we first examined the expression and localization of FBXL6. Our IHC staining of liver tissues from patients demonstrated that FBXL6 was expressed mainly in HCC cells (Fig. 1a). To extend this work, we analyzed the relationship between FBXL6 expression and clinical prognosis in HCC patients. IHC staining of 108 paired HCC samples (tumor tissues and adjacent normal tissues from the same patients) revealed that FBXL6 was overexpressed in 60.2% (65/108) of the HCC tissues compared to the paired adjacent tissues and was positively associated with advanced TNM stage, vascular thrombosis, and metastasis (Fig. 1b, Supplementary Table 1). In line with this finding, FBXL6 exhibited high expression in 94% of HCC tissues compared to the matched adjacent normal tissues in The Cancer Genome Atlas (TCGA) datasets (Supplementary Fig. 1a). Furthermore, we investigated the mutation profiles for the FBXL6-overexpressing HCC cases and found frequent TP53 or CTNNB1 mutation in this subset of HCC patients (Supplementary Fig. 1b). Univariate and multivariate analyses showed that FBXL6 was strongly associated with overall survival (OS) in HCC patients, supporting the idea that FBXL6 expression is an independent risk factor in HCC (Fig. 1c, Supplementary Table 2). Thus, these results indicate that elevated FBXL6 expression is positively associated with metastasis in HCC patients.

To address the role of FBXL6 in HCC metastasis in vivo, we generated Fbxl6^LSL-fl/+^ mice by inserting the CAG promoter-LoxP-STOP-LoxP-mouse Fbxl6 cDNA cassette into the ROSA26 locus in C57BL/6 N mice by CRISPR/Cas9-mediated genome editing. *Fbxl6;Alb-Cre* mice were obtained by crossing *Fbxl6*^*LSL-fl/+*^ mice with *Alb-Cre* mice (Supplementary Fig. 2a), and their *Alb-Cre* (wild-type) littermates were monitored for tumorigenesis and metastasis for 310 days, at which time they were imaged. The mouse genotype was validated by PCR (Supplementary Fig. 2b). We found that elevated expression of FBXL6 in hepatocytes was very strongly related to hepatocarcinogenesis and lung metastasis. The promotive effects on metastasis in these mice were much stronger than those in mice with Kras mutation (*Kras*^*LSL-G12D/+*^*;Alb-Cre*), p53 haploinsufficiency (*p53*^*+/*–^) or Tsc1 insufficiency (*Tsc1*^*fl/fl*^*;Alb-Cre*), as indicated by the tumor number, largest tumor size, liver/body weight ratio, and rate of lung metastasis (Fig. 1d–h). Furthermore, there were no significant differences between female and male mice in the number of FBXL6-induced tumors, largest tumor size and liver/body weight ratio, suggesting that the oncogenic activity of FBXL6 is independent of sex (Supplementary Fig. 2c). H&E staining showed that in *Fbxl6;Alb-Cre* mice that developed spontaneous liver tumors, the tumors formed within blood vessels, while in *Alb-Cre* mice, no such liver tumors formed (Supplementary Fig. 3a). The presence of abnormal cells around the vessels was significantly increased in *Fbxl6;Alb-Cre* mice compared with *Alb-Cre* mice (Supplementary Fig. 3b). Importantly, FBXL6 mice with elevated FBXL6 expression developed spontaneous liver tumors and lung metastasis (Fig. 1i, j), as indicated by the number of metastases in FBXL6-overexpressing mice compared to wild-type mice (Fig. 1k). In line with this finding, the mRNA expression of metastasis markers (*Icam1, Vcam1, Upa, Ccl2*, and *Mmp9*)^30^ was elevated in the liver tumors of mice with high *Fbxl6* expression compared with the liver tissues of wild-type mice (Fig. 1l). H&E staining of lung metastases and IHC staining of the hepatocyte marker Lipase C (LIPC) further validated the FBXL6-driven lung metastasis of HCC (Fig. 1m). In line with these findings, FBXL6 promoted HCC cell migration and upregulation of metastasis markers (*Icam1, Vcam1, Upa, Ccl2*, and *Mmp9*) (Supplementary Fig. 3c, d), while silencing FBXL6 blocked these effects (Supplementary Fig. 3e–g). Taken together, these findings indicate that via elevated expression, FBXL6 acts as a superoncogene in driving HCC development in vivo and in vitro.

### Characterization of the proteome and ubiquitinome in response to FBXL6 overexpression

To systematically identify the substrates of FBXL6, we carried out mass spectrometry-based label-free quantitative proteomics and ubiquitomics on FBXL6-high HCC tumor tissues and adjacent tissues from *Fbxl6;Alb-Cre* mice (Supplementary Fig. 4a). Through these analyses, we identified 4384 proteins and 3198 quantifiable proteins. We defined candidate proteins with significant differential expression (*p* < 0.05 by Student’s t test) as those with a fold change of at least 1.2 between FBXL6-induced HCC tumors and adjacent tissues. With this criterion, 851 downregulated proteins and 1123 upregulated proteins in HCC tumors with high FBXL6 expression were identified (Supplementary Fig. 4b).

FBXL6 overexpression caused a global increase in protein ubiquitination after normalization to the total protein content (Supplementary Fig. 4c). In total, 1710 ubiquitinated sites were quantified for the ubiquitinomics analysis. Based on a criterion of a ≥1.5-fold increase in ubiquitination in FBXL6-overexpressing HCC tumors compared to adjacent tissues, a total of 500 significantly upregulated ubiquitin sites in 296 proteins were identified in FBXL6-overexpressing HCC tumors. A summary of the results of these analyses is shown in Supplementary Fig. 4c.

### FBXL6 triggers K63-linked TKT ubiquitination at Lys16(K16) and Lys319 (K319), leading to TKT activation and HCC metastasis

**Fig. 2:**
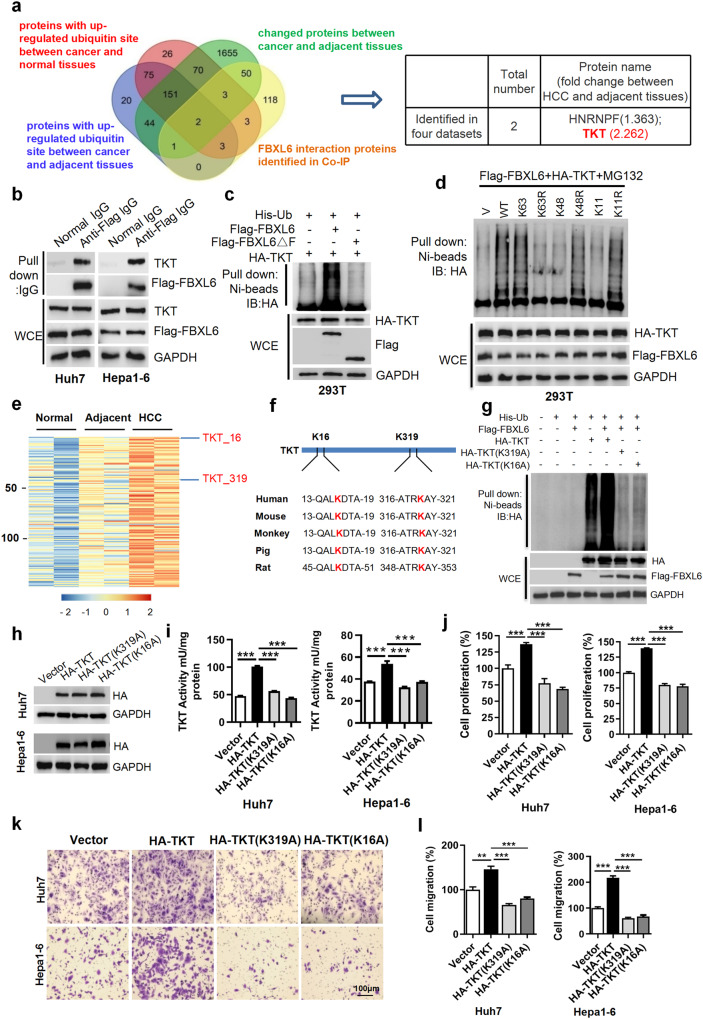
FBXL6 triggers K63-linked TKT ubiquitination at K16 and K319, leading to TKT activation and HCC metastasis. **a** Diagram showing the number of proteins with an expression fold change of more than 1.2 between cancer and adjacent tissues (green), proteins with increased ubiquitin sites (a cutoff of a ≥ 1.5-fold change; blue/red), and proteins binding to FBXL6 (yellow) in HCC tumors. **b** FBXL6 bound to endogenous TKT. Human Huh7 and mouse Hepa1-6 cells were transfected with Flag-FBXL6 plasmids for 48 h and were then lysed. The indicated antibodies and Protein A/G PLUS-Agarose were added to the cell lysates. **c** HEK293T cells were cotransfected with the Flag-FBXL6ΔF (deletion of F-box domain), Flag-FBXL6, or HA-TKT plasmid and the ubiquitin plasmid for 45 h and were then exposed to MG132 (10 µM) for 3 h to block protein degradation. Cells were lysed in RIPA buffer, followed by pulldown using Ni-NTA beads or direct immunoblotting (IB) with the indicated antibodies. **d** HEK293T cells were cotransfected with the Flag-FBXL6 and HA-TKT plasmids and the indicated ubiquitin plasmids, followed by pulldown using Ni-NTA beads or direct IB with the indicated antibodies. K63, K48, and K11, indicating Lys residues in ubiquitin, were individually mutated to Arg. **e** Heatmap showing the 147 proteins with increased ubiquitination in HCC tissues compared to adjacent tissues and in adjacent tissues compared to normal tissues. **f** Examination of the TKT protein sequence identified K319 and K16 in TKT as evolutionarily conserved in different species. **g** HEK293T cells were cotransfected with the Flag-FBXL6 plasmid and the wild-type TKT or a TKT mutant plasmid for 45 h and were then treated with MG132 (10 µM) for 3 h to block protein degradation. Cells were lysed in RIPA buffer, followed by pulldown using Ni-NTA beads or direct Western blotting with the indicated antibodies. **h**–**l** Huh7 or Hepa1-6 cells were transfected with HA-TKT, HA-TKT (K319A), or HA-TKT (K16A) for 48 h. A portion of the cells was harvested for Western blotting (**h**) or detection of TKT activity using a TKT-specific activity assay kit (**i**). Another portion was used for a cell proliferation assay (**j**). Other portions were reseeded into Transwell plates for a migration assay (**k**, **l**). Data are representative of three independent experiments. Scale bar, 100 μm. One-way ANOVA with Tukey’s multiple comparisons test was used. *n* = 3–4, biological replicates. **p ≤ 0.01, ***p ≤ 0.001.

To further screen the candidate FBXL6 substrates, we reanalyzed protein expression and ubiquitination in HCC tumors and adjacent tissues using proteomics and ubiquitomics analyses, respectively. A total of 231 proteins were ubiquitinated in HCC tumors but not in the adjacent tissues (a cutoff of a 1.5-fold change in the ubiquitination level). A total of 1974 proteins showed significant differential expression (a cutoff of a ≥1.2-fold change in the protein level) between HCC and adjacent tissues. Furthermore, 180 proteins were identified as candidate interacting proteins with FBXL6 in a pulldown assay using an anti-FBXL6 antibody in HCC cells. We made a chart showing the overlapping proteins to identify potential FBXL6 substrates and identified 2 overlapping proteins, HNRNPF (heterogeneous nuclear ribonucleoprotein F) and TKT (transketolase). Both of these proteins exhibited high levels of ubiquitination and changes in protein expression (Fig. 2a), suggesting that they might be substrates of FBXL6. TKT, a key enzyme in the nonoxidative branch of the pentose phosphate pathway (PPP), was upregulated 2.262-fold in HCC tissues compared to adjacent normal tissues (Fig. 2a)^2,22^, whereas HNRNPF was upregulated only 1.363-fold in HCC tissues compared to adjacent normal tissues. Further, compared to knockdown of HNRNPF, knockdown of TKT dramatically inhibited cell proliferation and migration (Supplementary Fig. 5a–c), suggesting that TKT but not HNRNPF may play a critical role in FBXL6-driven HCC cell migration. To test the interaction between FBXL6 and TKT, we performed a co-IP experiment and found that FBXL6 was able to interact with TKT, whereas the previously reported potential substrates of FBXL6 (HSP90, CCNA2, and VDAC2) had low or no binding affinity compared with that of TKT in HCC cells (Fig. 2b, Supplementary Fig. 6a, b). These findings suggest that TKT binds to FBXL6. To further confirm whether FBXL6 ubiquitinates TKT, we cotransfected HA-TKT and his-ubiquitin with Flag-FBXL6 or Flag-FBXL6ΔF (a mutant with deletion of the F-box domain) into 293 T cells and then treated the cells with MG132 for 3 h before performing an in vivo ubiquitination assay. Overexpression of FBXL6 increased the level of ubiquitinated TKT, whereas no such effect was observed after overexpression of the F-box deletion mutant of FBXL6 (FBXL6ΔF) (Fig. 2c). To strengthen these findings, we performed domain analyses to further characterize the FBXL6-TKT interaction and TKT ubiquitination. We reconstructed FBXL6 mutants with deletion of the leucine-rich repeat (LRR) domain: FBXL6(ΔLRR219-244), FBXL6(ΔLRR383-408) and FBXL6(ΔLRR497-528) (Supplementary Fig. 6c). The results of the co-IP experiment indicated that deletion of LRR497-528 (ΔLRR497-528) in FBXL6 significantly attenuated the interaction between TKT and FBXL6 compared with that in cells expressing wild-type FBXL6 or the other deletion mutants (ΔLRR383-408 and ΔLRR219-244), suggesting that LRR497-528 in FBXL6 is responsible for the FBXL6-TKT interaction (Supplementary Fig. 6d). As expected, compared with wild-type FBXL6 and the other deletion mutants, FBXL6(ΔLRR497-528) lost the ability to promote TKT polyubiquitination, a finding that further indicates that FBXL6 ubiquitinates TKT (Supplementary Fig. 6e). SCF (Skp1-Cullin-F box) family members usually catalyze Lys63-linked polyubiquitination of a target protein to induce its activation^31^, whereas Lys48- or Lys11-linked polyubiquitination induces proteasomal degradation of the target protein^32,33^. We thus generated three ubiquitin mutants, ubiquitin-K63R, ubiquitin-K48R, and ubiquitin-K11R (in which K63, K48, or K11 was substituted with arginine), to determine the type of ubiquitin linkage involved in FBXL6-mediated TKT ubiquitination. Mutation of K63 (K63R) in ubiquitin nearly completely abolished FBXL6-mediated ubiquitination of TKT, whereas the K48R and K11R mutations in ubiquitin, like wild-type ubiquitin, significantly promoted TKT polyubiquitination, indicating that FBXL6 catalyzes K63-linked polyubiquitination of TKT (Fig. 2d).

Whether posttranslational regulation of TKT is associated with its activity is unknown. Our ubiquitomics analysis indicated that TKT ubiquitination at Lys16 (K16) and Lys319 (K319) was significantly increased in HCC tumors (Fig. 2e). We further examined the TKT protein sequence and found that these two lysine sites were evolutionarily conserved (Fig. 2f). To confirm that K319 and K16 in TKT are ubiquitinated by FBXL6, we generated two mutants, namely, K319A and K16A, to disrupt the ubiquitination of TKT at K319 and K16, respectively. Interestingly, the ubiquitination-disrupting K319A and K16A mutants exhibited a significant loss of ability to be ubiquitinated by FBXL6, suggesting that FBXL6 promotes K63-linked TKT ubiquitination at K319 and K16 (Fig. 2g). To confirm whether these two lysine residues in TKT are related to its activation, we analyzed TKT activity using a TKT-specific activity assay kit. Wild-type TKT had high activity, whereas the ubiquitination-disrupting mutants (K319A and K16A) exhibited a complete loss of activity in both human and mouse hepatocellular carcinoma cells (Fig. 2h, i). Consistent with these findings, both the TKT-K319A and TKT-K16A mutants significantly attenuated NADPH production compared with wild-type TKT, as indicated by the NADPH/NADP^+^ ratio (Supplementary Fig. 7a). These results demonstrated that the K319 and K16 residues are associated with TKT activation. Previous studies reported that TKT promotes cancer cell proliferation and migration. As expected, the ubiquitination-disrupting mutants (K319A and K16A) lost the ability to promote the proliferation and migration of both human and mouse hepatocellular carcinoma cells (Fig. 2j–l). To determine whether TKT-mediated cell proliferation and migration depend on FBXL6, we cotransfected FBXL6 with TKT or a TKT mutant (K319A or K16A) into Huh7 and Hepa1-6 cells. FBXL6 significantly upregulated wild-type TKT activity and promoted TKT-mediated cell proliferation and migration but did not affect the TKT mutants (K319A and K16A) (Supplementary Fig. 7b–e), suggesting that FBXL6 activates TKT via K63-linked polyubiquitination at K319 and K16. Furthermore, wild-type TKT was localized mainly in the cytoplasm, whereas its mutants (K319A and K16A) accumulated in the nucleus (Supplementary Fig. 8a, b), suggesting that ubiquitinated TKT localizes mainly in the cytoplasm.

To further study the effect of TKT on FBXL6-triggered HCC migration, we reduced TKT expression in *FBXL6;Alb-Cre* and *Alb-Cre* primary hepatocytes and FBXL6-overexpressing HCC cells using TKT siRNAs. FBXL6 promoted HCC cell proliferation and migration, and knockdown of TKT dramatically attenuated these effects (Supplementary Fig. 9a–c). Furthermore, TKT-mediated HCC cell proliferation and migration were dependent on FBXL6, as indicated by the finding that knockdown of FBXL6 attenuated the oncogenic effects of TKT (Supplementary Fig. 9d, e). Collectively, these results indicate that FBXL6 triggers the K63-linked polyubiquitination, cytoplasmic localization, and activation of TKT, leading to HCC metastasis.

### TKT phosphorylation at Thr287 is critical for the activation and cytoplasmic localization of TKT and the interaction of TKT with the E3 FBXL6

**Fig. 3:**
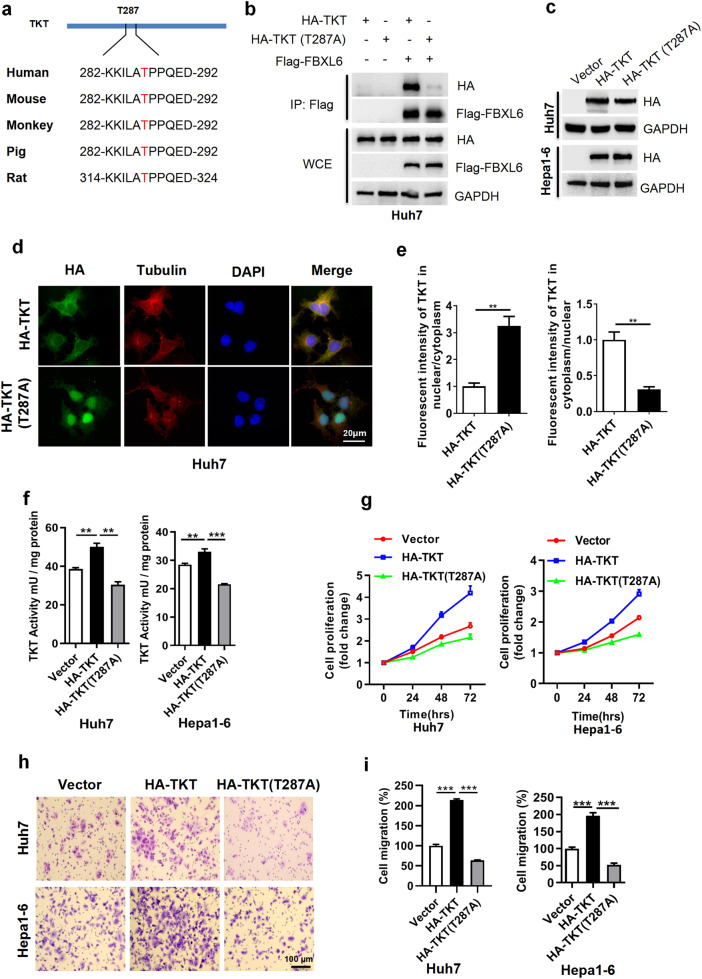
TKT phosphorylation at Thr287 is critical for TKT activation, cytoplasmic localization, and TKT-E3 ligase FBXL6 interaction. **a** Examination of the TKT protein sequence identified Thr287 in TKT as evolutionarily conserved in different species. **b** FBXL6 binds to wild-type TKT but not its mutant (T287A). Huh7 cells were transfected with the indicated plasmids for 48 h, followed by pulldown with anti-Flag IgG. **c–i** Huh7 and Hepa1-6 cells were transfected with the wild-type TKT or its TKT T287A mutant plasmid for 48 h. A portion of the cells was harvested for Western blotting (**c**). Another portion was used for staining TKT and its mutant (T287A) (**d**, **e**), or detection of TKT activity using a TKT activity assay kit (**f**). Other portions were replated into 96-well plates for a cell proliferation assay (**g**) or into Transwell plates and incubated overnight for a migration assay (**h**, **i**). The nuclear and cytoplasmic fluorescence intensity in the cells described in (**d**) was determined by ImageJ software. Scale bar, 20 μm. Unpaired *t* test was used in (**e**); one-way ANOVA with Tukey’s multiple comparisons test was used in (**f**, **i**). **p ≤ 0.01, ***p ≤ 0.001.

FBXL6 is an F-box SCF-E3 that forms a functional complex with Cullin-1, Skp1, and Rbx1 proteins. F-box proteins usually specifically recognize and bind potential substrates in a manner dependent on phosphorylation^34^. Therefore, it is reasonable to hypothesize that the TKT protein needs to be phosphorylated before it can be recognized by FBXL6. To investigate this possibility, the changes in phosphorylated TKT in FBXL6-overexpressing tumors were analyzed using a phosphoproteomics approach. An increase in TKT phosphorylation at Thr287 was observed in liver tumors (Supplementary Fig. 10a). Previous literature indicated that AKT-mediated TKT phosphorylation at Thr382 is associated with its activation in mice fed a lysine-deficient diet^35^, whereas we found that only Thr287 in TKT was phosphorylated in HCC tumors with high FBXL6 expression, indicating that TKT phosphorylation at Thr287 may play a critical role in FBXL6-mediated TKT ubiquitination. Furthermore, this site was highly evolutionarily conserved in various species (Fig. 3a). To investigate whether this site is responsible for the FBXL6-TKT interaction, we cotransfected Huh7 cells with a TKT mutant (TKT-Thr287A) and FBXL6 and performed a co-IP experiment. Interestingly, FBXL6 pulled down wild-type TKT, but this interaction was completely disrupted by Thr287 mutation in TKT, suggesting that phosphorylation of TKT at Thr287 is critical for the interaction between FBXL6 and TKT (Fig. 3b).

To determine whether Thr287 phosphorylation is associated with the cellular localization of TKT, we detected TKT by immunofluorescence staining and found that the TKT T287A mutant was localized mainly in the nucleus, whereas wild-type TKT was localized in the cytoplasm (Fig. 3c–e). To determine whether Thr287 phosphorylation is responsible for TKT activation, we analyzed the activity of the TKT T287A mutant using the TKT activity assay kit. The Thr287 mutant exhibited reduced TKT activity compared with wild-type TKT (Fig. 3c, f). In line with this finding, TKT T287A blocked cell proliferation and migration compared with wild-type TKT (Fig. 3g–i). Taken together, these findings indicate that Thr287 is the critical amino acid for the interaction between TKT and FBXL6 and for TKT activation.

### VRK2 is important for TKT phosphorylation at Thr287 and facilitates FBXL6-mediated K63-linked ubiquitination of TKT and TKT activation

**Fig. 4:**
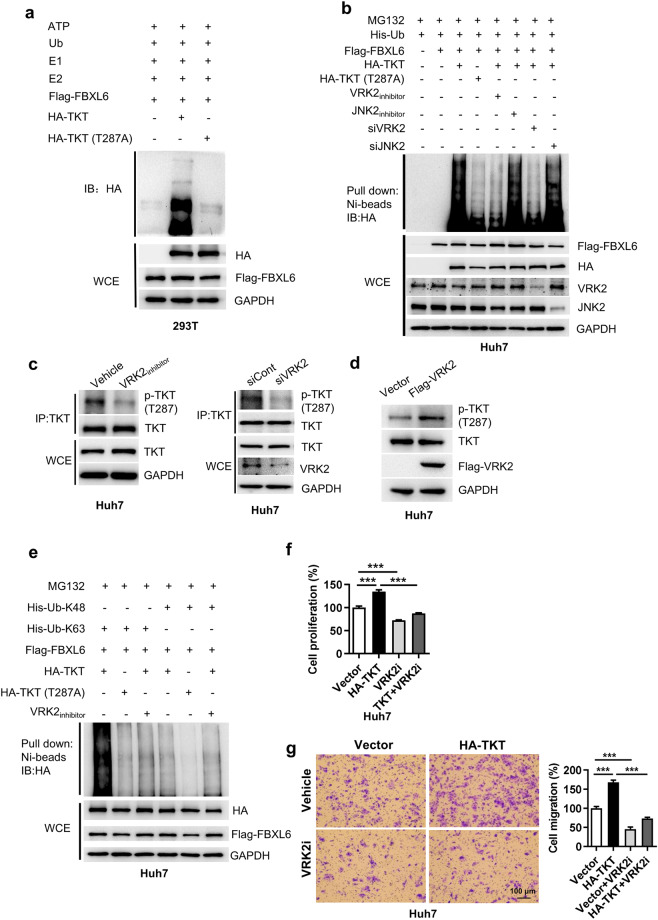
VRK2 is important for TKT phosphorylation at Thr287 and facilitates FBXL6-mediated TKT K63-linked ubiquitination and activation. **a** HEK293T cells were transfected with HA-TKT and its mutant T287A, followed by IP purification and in vitro ubiquitination. Ubiquitinated TKT was detected with an anti-HA antibody. **b** Huh7 cells were transfected with the Flag-FBXL6 plasmid and the HA-TKT or HA-TKT (T287A) plasmid in the presence or absence of a CK1/VRK2 inhibitor (IC261, 5 μM), a JNK2 inhibitor (JNK inhibitor IX, 0.5 μM), siRNA targeting VRK2 (siVRK2) or siJNK2, followed by pulldown using Ni-NTA beads or direct IB with the indicated antibodies. **c** Inhibition or knockdown of VRK2 reduced TKT phosphorylation at Thr287. Huh7 cells were treated with a CK1/VRK2 inhibitor (IC261, 5 μM) or siVRK2 for 48 h and were then subjected to a pulldown assay using an anti-TKT antibody. **d** Huh7 cells were transfected with Flag-VRK2 for 48 h and were then harvested for Western blotting with an anti-pTKT (Thr287) antibody. **e** Huh7 cells were cotransfected with the Flag-FBXL6 plasmid and the HA-TKT or HA-TKT (T287A) plasmid with the indicated ubiquitin plasmids in the presence or absence of CK1/VRK2 inhibitor (IC261, 5 μM), followed by pulldown using Ni-NTA beads or direct IB with the indicated antibodies. **f**, **g** Huh7 cells were transfected with Flag-FBXL6 and treated with or without a CK1/VRK2 inhibitor (IC261, 5 μM) for 48 h and were then reseeded into 96-well plates for a cell proliferation assay (**f**) or into Transwell plates for a migration assay (**g**). *n* = 3–6, biological replicates. Scale bar, 100 μm. One-way ANOVA with Tukey’s multiple comparisons test was used. ***p ≤ 0.001.

We next examined whether Thr287 is involved in FBXL6-mediated TKT ubiquitination. The results of the in vitro ubiquitination assay showed that FBXL6 promoted TKT ubiquitination, and this effect was attenuated by the TKT T287A mutation (Fig. 4a), suggesting that Thr287 plays an important role in FBXL6-mediated TKT polyubiquitination. Subsequently, we investigated the potential kinases involved in Thr287 TKT phosphorylation using GPS 2.0 software^36^. VRK2 and JNK2 were identified as the top two candidates that may phosphorylate TKT at T287 (Supplementary Fig. 10b). The in vivo ubiquitination assay results showed that inhibition or knockdown of VRK2 but not JNK2 significantly attenuated FBXL6-mediated TKT polyubiquitination (Fig. 4b). This effect was similar to that of the TKT T287A mutation (Fig. 4b). To further confirm whether TKT is phosphorylated at T287 by VRK2, we analyzed the effect of VRK2 inhibition or knockdown on TKT phosphorylation by Western blotting. We found that inhibition or knockdown of VRK2 significantly blocked TKT phosphorylation at T287 (Fig. 4c). Conversely, overexpression of VRK2 promoted phosphorylation of TKT at Thr287 (Fig. 4d). These findings indicated that TKT Thr287 is a VRK2 phosphorylation site. Inhibition of VRK2 or overexpression of the TKT mutant (T287A) dramatically blocked FBXL6-mediated K63-linked TKT polyubiquitination (Fig. 4e). We next examined the role of VRK2 in TKT-mediated cell proliferation and migration. Inhibition of VRK2 significantly attenuated TKT-mediated cell proliferation and migration (Fig. 4f, g). Taken together, these data demonstrate that VRK2 is involved in the phosphorylation of TKT at T287, which is required for FBXL6-mediated TKT K63-linked polyubiquitination and activation.

### FBXL6 upregulates PD-L1 and VRK2 expression via a TKT-dependent decrease in ROS accumulation and mTOR activation, leading to immune evasion and HCC metastasis

**Fig. 5:**
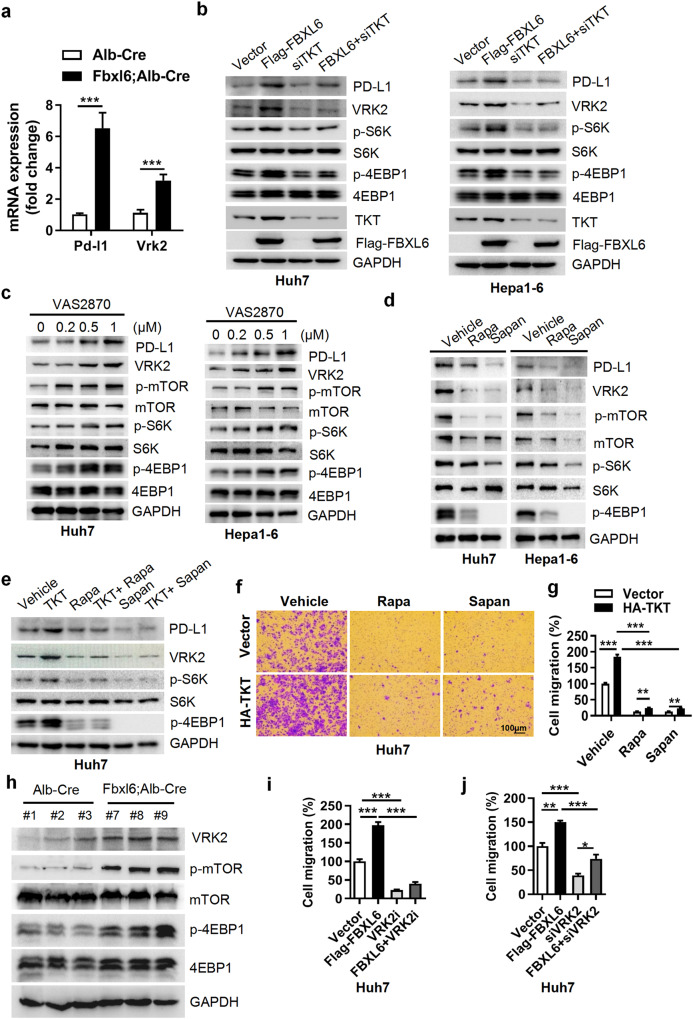
FBXL6 upregulates PD-L1 and VRK2 expression via a TKT-dependent decrease in ROS accumulation and mTOR activation, leading to immune evasion and HCC metastasis. **a** PD-L1 and VRK2 are upregulated in FBXL6 tumors. PD-L1 and VRK2 mRNA levels were measured by qPCR in normal tissues and FBXL6 tumors. **b** Huh7 and Hepa1-6 cells were transfected with the indicated plasmids or siRNA oligos for 48 h. PD-L1, VRK2, and mTOR downstream targets (p-S6K and p-4EBP1) were analyzed by Western blotting. **c** VRK2 and mTOR signaling pathways were assessed after 48 h of treatment with the ROS inhibitor VAS2870 in Huh7 and Hepa1-6 cells. **d** Huh7 or Hepa1-6 cells were treated with mTOR inhibitors (rapamycin, Rapa (100 nM); sapanisertib, Sapan (0.5 μM)) for 48 h and were then harvested for Western blotting. **e**–**g** Huh7 cells were transfected with HA-TKT in the presence or absence of mTOR inhibitors (Rapa (100 nM); Sapan (0.5 μM)) for 48 h. A portion of the cells was harvested for Western blotting (**e**). The other portion of the cells was seeded into Transwell plates overnight for a migration assay (**f**, **g**). Migration was quantified after crystal violet (0.5% w/v) staining. **h** VRK2, p-mTOR, and p-4EBP1 protein levels were analyzed in FBXL6-overexpressing tumors. **i**, **j** Huh7 cells were transfected with FBXL6 plasmids and transfected with an siRNA targeting VRK2 or treated with a VRK2 inhibitor for 48 h. The cells were reseeded into Transwell plates and incubated overnight, and migration was quantified after crystal violet (0.5% w/v) staining. Unpaired *t* test was used in (**a**). One-way ANOVA with Tukey’s multiple comparisons test was used in (**i**, **j**). Two-way ANOVA with the Bonferroni correction for multiple comparisons was used in (**g**). *p ≤ 0.05, **p ≤ 0.01, ***p ≤ 0.001.

The immune microenvironment plays an important role in tumorigenesis and metastasis. Here, we found that PD-L1 was highly expressed in FBXL6-high tumors compared with normal tissues (Fig. 5a), suggesting that PD-L1 may be a downstream target of the FBXL6-TKT axis. To validate this hypothesis, we cotransfected FBXL6 with a siRNA targeting TKT in Huh7 and Hepa1-6 cells and found that FBXL6 upregulated PD-L1 expression and that knockdown of TKT blocked this effect (Fig. 5b). A previous study reported that TKT reduced intercellular ROS accumulation. Here, we found that the TKT-mediated decrease in ROS accumulation was significantly blocked by knockdown of FBXL6 (Supplementary Fig. 11a–c). This finding further confirms that TKT activity depends on FBXL6. Intercellular ROS scavenging can activate the mTOR pathway^37,38^. We found that treatment with the ROS inhibitor VAS2870 significantly increased the protein levels of PD-L1 and p-mTOR (whose downstream targets are p-S6K and p-4EBP1) and attenuated the TKT knockdown-mediated decreases in cell proliferation and migration (Fig. 5c, Supplementary Fig. 11d, e). Next, we sought to determine whether there is a positive correlation between mTOR activation and PD-L1 expression. Inhibition of mTOR with its inhibitors (rapamycin and sapanisertib) significantly reduced PD-L1 expression and TKT-mediated HCC cell proliferation and migration (Fig. 5d–g, Supplementary Fig. 11f). Taken together, these findings indicate that the FBXL6-TKT-ROS-mTOR axis positively regulates PD-L1 expression.

High expression of VRK2 was observed in FBXL6-overexpressing liver tumors (Fig. 5a). VRK2 is often highly expressed in various types of cancers and is involved in cancer pathogenesis, but the underlying mechanisms remain unclear. We sought to determine whether the FBXL6-TKT-mediated ROS-mTOR pathway regulates VRK2 expression. To this end, Huh7 and Hepa1-6 hepatocellular cancer cells were cotransfected with FBXL6 with or without a siRNA targeting TKT. FBXL6 transfection upregulated VRK2 expression, and silencing TKT attenuated VRK2 expression (Fig. 5b). Furthermore, treatment with the ROS inhibitor VAS2870 upregulated VRK2 expression, and treatment with the mTOR inhibitors significantly reduced VRK2 expression (Fig. 5c–e). In line with these findings, mTOR and VRK2 signaling was upregulated in FBXL6 tumors compared with normal tissues (Fig. 5h). Inhibition or knockdown of VRK2 significantly blocked the FBXL6-mediated cell proliferation and migration of HCC cells (Fig. 5i, j, Supplementary Fig. 11g, h). Taken together, these findings demonstrate that VRK2 is a downstream target of the FBXL6-TKT-ROS-mTOR axis and facilitates FBXL6-mediated HCC tumorigenesis and metastasis.

### Targeting or interfering with TKT suppresses HCC tumorigenesis and lung metastasis triggered by FBXL6 in vivo

**Fig. 6:**
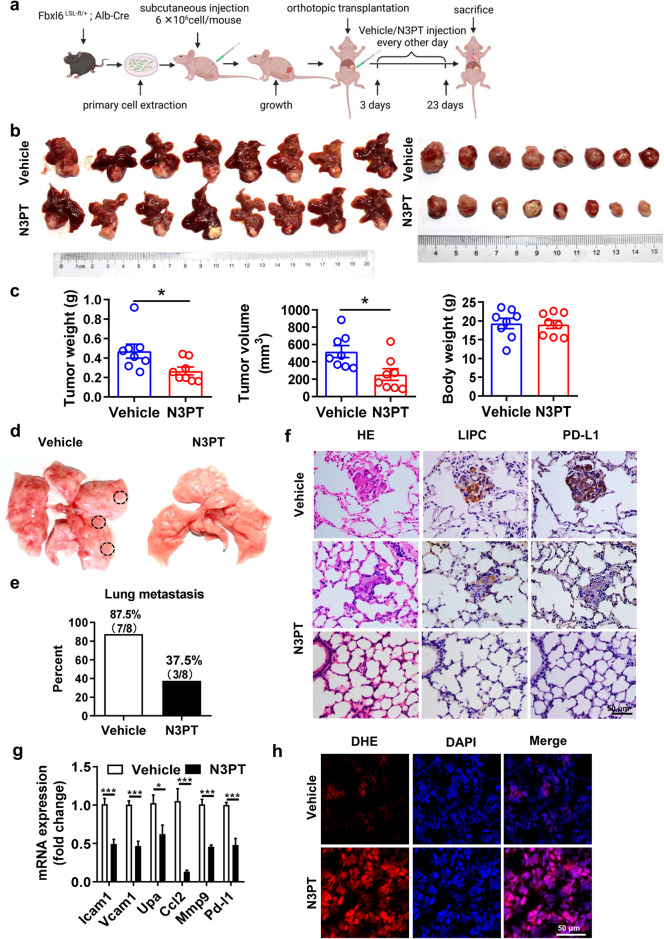
Inhibition of TKT significantly blocks HCC tumorigenesis and lung metastasis triggered by FBXL6 in vivo. **a** Schematic diagram showing the established orthotopic HCC model in nude mice and the pharmacological treatment plan. **b** Photographs of whole livers with orthotopic tumors (left) and isolated tumors (right) from the vehicle (PBS) group (*n* = 8) and N3PT group (*n* = 8). **c** Effects of N3PT on the weight and volume of FBXL6-overexpressing tumors in mice. The tumor volume was calculated using the following equation: tumor volume=3/4×π×a×b^2^ (where a is the longer diameter of the tumor and b is the shorter diameter of the tumor). The weight of the animals was monitored, and the results were plotted; *n* = 8. **d** Representative images showing the effect of N3PT on lung metastasis. **e** The lung metastasis rate was lower in the N3PT group (37.5%, 3/8) than in the control group (87.5%, 7/8). **f** Representative H&E and IHC staining images showing the low formation rate of distinct lung metastatic foci expressing the hepatocytic marker lipase C (LIPC) and PD-L1 after N3PT treatment. Scale bar, 50 µm. **g** The expression of metastasis-related genes (*Icam1*, *Vcam1*, *Upa*, *Ccl2*, and *Mmp9*) and *Pd-l1* was analyzed by qPCR; *n* = 6. **h** The ROS levels in liver tumors of N3PT- and vehicle-treated mice were evaluated by staining with DHE. Scale bar, 50 μm. Two-tailed Student’s unpaired *t* test was used in (**c**). Two-way ANOVA with the Bonferroni correction for multiple comparisons was used in (**g**). *p ≤ 0.05, ***p ≤ 0.001.

To study whether inhibition of TKT blocks FBXL6-triggered HCC lung metastasis, FBXL6 primary cells were cultured, transplanted into the hypodermis of nude mice, and then retransplanted into the livers of 16 male C57BL/6 N mice. The process for establishing this orthotopic HCC model in mice and the pharmacological treatment plan are shown in Fig. 6a. Compared to vehicle treatment, treatment with N3PT (25 mg/kg, i.p., every other day), a TKT inhibitor^39^, significantly reduced the tumor weight and tumor volume (Fig. 6b, c). Consistent with this finding, knockdown of TKT significantly attenuated tumor growth in *Fbxl6;Alb-Cre* mice, as characterized by the decreased tumor number and tumor size (Supplementary Fig. 12a-c). Importantly, N3PT treatment significantly blocked HCC lung metastasis, as indicated by the decreased rate of lung metastasis in the N3PT group compared with the control group (Fig. 6d, e). To confirm the distant lung metastasis of HCC, H&E and IHC analyses of metastatic nodules were performed. The number of lung metastatic nodules labeled with the hepatocyte marker lipase C was significantly increased in the control group compared with the N3PT treatment group (Fig. 6f). In line with these findings, knockdown of TKT significantly reduced HCC lung metastasis in *Fbxl6; Alb-Cre* mice, as indicated by the decreases in the lung metastasis rate and lipase C expression in metastatic nodules (Supplementary Fig. 12d, e). Notably, PD-L1 expression was significantly reduced after N3PT treatment, indicating that inhibition of TKT attenuates immune evasion (Fig. 6f, g). Consistent with this finding, the expression of metastasis markers (*Icam1, Vacm1, Upa, Ccl2*, and *Mmp9*) was significantly reduced in the N3PT treatment group compared to the control group (Fig. 6g). To extend this work, the effect of N3PT on ROS accumulation in these two groups was examined. N3PT significantly attenuated the FBXL6-mediated decrease in ROS accumulation in liver tumors (Fig. 6h). Furthermore, N3PT reduced the expression of the proliferation biomarkers Ki67 and CCNB2 and the metastasis biomarker MMP9 in liver tissues (Supplementary Fig. 13a). To further confirm the efficacy of N3PT in a mouse model, we tested the effect of the TKT inhibitors N3PT and oxythiamine (OT) on HCC cells in vitro. N3PT and OT significantly blocked the FBXL6-mediated proliferation of HCC cells (Huh7 and Hepa1-6) (Supplementary Fig. 13b). Taken together, these results indicate that inhibition of TKT constitutes a potential therapeutic strategy for HCC tumorigenesis and lung metastasis driven by abnormally high FBXL6 expression.

### The activated TKT protein level correlates positively with the protein levels of FBXL6 and VRK2 and predicts unfavorable survival in HCC patients

**Fig. 7:**
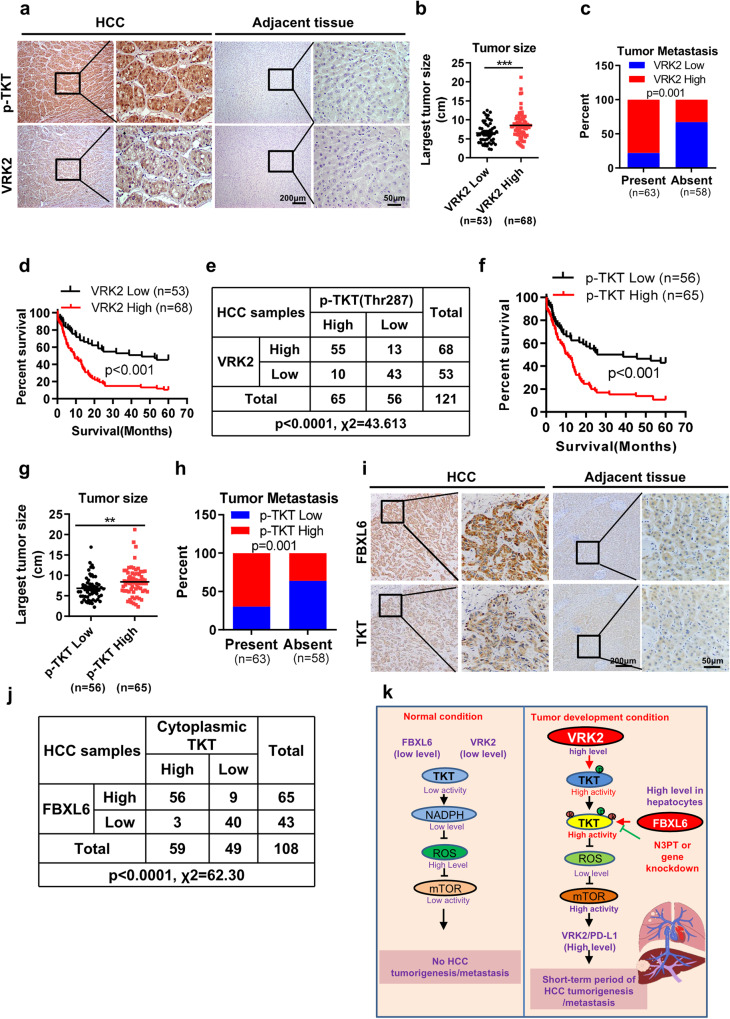
The activated TKT protein level correlates positively with the protein levels of FBXL6 and VRK2 and predicts unfavorable survival in HCC patients. **a** Tissue sections of adjacent normal or HCC tissue from patients were subjected to IHC staining for p-Thr287 TKT and VRK2. Representative images are shown. Scale bar, 200 μm or 50 μm. **b**, **c** High expression of VRK2 was positively associated with tumor size and metastasis. **d** The prognostic significance of VRK2 in HCC patients was evaluated by Kaplan–Meier analysis. High expression of VRK2 predicted a shorter overall survival time. **e** The association between the p-Thr287 TKT and VRK2 protein levels was evaluated by the χ2 test in 121 HCC tissues. **f** The prognostic significance of p-Thr287 TKT in HCC patients was evaluated by Kaplan–Meier analysis. **g**, **h** A high level of p-Thr287 TKT was positively associated with tumor size and metastasis. **i** Tissue sections of adjacent normal or HCC tissue from patients were subjected to IHC staining for cytoplasmic TKT and FBXL6. Representative images are shown. Scale bars, 200 μm or 50 μm. **j** The association between cytoplasmic TKT and FBXL6 was evaluated by the χ2 test in 108 HCC tissues. **p ≤ 0.01, ***p ≤ 0.001. **k** A model of FBXL6-driven HCC tumorigenesis and metastasis in vivo. Under physiological conditions, hepatocytes with low levels of FBXL6 and VRK2 expression have lower levels of TKT phosphorylation and ubiquitination; thus, TKT activity is maintained at a low level. Upon FBXL6 upregulation in hepatocytes, TKT is first phosphorylated at Thr287 by VRK2. The phosphorylated TKT is then recognized and ubiquitinated at K16/319 by SCF-FBXL6, which promotes TKT activation and cytoplasmic localization. Activated TKT can upregulate VRK2 and PD-L1 expression through a decrease in ROS accumulation and activation of mTOR, leading to immune escape and HCC metastasis.

Based on our finding that VRK2 phosphorylation promotes TKT activation in mice, we investigated the expression level of VRK2 in HCC patients. VRK2 was highly expressed in HCC tumors (Fig. 7a). Increased VRK2 expression levels were positively associated with advanced tumor stage, recurrence, vascular thrombosis, and metastasis (Fig. 7b, c; Supplementary Table 3). In line with this finding, a higher VRK2 expression level correlated with a shorter overall survival (OS) time in 121 HCC patients (Fig. 7d; hazard ratio = 0.3634, *p* < 0.001) and was identified as an independent risk factor in HCC patients (Supplementary Table 4). Next, we tested the possible correlation between the activated TKT (pThr287-TKT antibody staining) and VRK2 protein levels. The level of activated TKT (p-Thr287) correlated positively with the VRK2 protein expression level (Fig. 7a, e; *p* < 0.0001). Furthermore, activated TKT (p-Thr287) was negatively associated with overall survival in HCC and correlated positively with TNM stage, recurrence, and metastasis (Fig. 7f–h, Supplementary Table 5). These data suggest that VRK2 and activated TKT (p-Thr287) may accurately predict clinical outcomes.

We next evaluated the association between activated cytoplasmic TKT and FBXL6 in HCC using patient tissues. First, we used IHC staining to evaluate TKT expression in HCC tissues. The level of cytoplasmic TKT was high in 54.6% (59/108) of the HCC tissues (Fig. 7i). High expression of FBXL6 was also observed in HCC tissues (Fig. 7i). A strong positive correlation between FBXL6 expression and the cytoplasmic TKT level was observed in 51.85% (56/108) of the HCC tissues (Fig. 7j). Importantly, high coexpression of FBXL6 and TKT in HCC tissues was associated with a worse prognosis than was low coexpression of these two proteins (Supplementary Fig. 14a). Consistent with this pattern, high coexpression of FBXL6 and TKT was associated with high-grade HCC, poorly differentiated tumors, and a high frequency of recurrence and metastasis compared with low coexpression of these two proteins (Supplementary Fig. 14b–e).

In conclusion, our study identifies a superoncogene named FBXL6 that plays a critical role in promoting HCC tumor formation and lung metastasis in mice and humans. Our results suggest that elevated FBXL6 expression promotes VRK2 phosphorylation-dependent TKT polyubiquitination and activation and thereby enhances ROS-mTOR-mediated upregulation of VRK2 and PD-L1 expression and immune evasion, ultimately promoting HCC metastasis (Fig. 7k). Targeting TKT could significantly block hepatocytic FBXL6-driven HCC metastasis.

## Discussion

Our findings identify FBXL6 expression as a previously unrecognized signature of liver tumorigenesis and lung metastasis. In particular, FBXL6 has been reported to regulate the cell development-related protein ETS transcriptional repressor Tel (ETV6) and its invertebrate ortholog Yan via ubiquitination and proteasomal degradation^40^. In addition, a recent study reported that FBXL6 promoted HSP90AA1 ubiquitination and thus enhanced the proliferation of HCC cells in vitro^14^. However, whether FBXL6 promotes liver cancer metastasis in vivo and the mechanism underlying this action remain unknown. In this study, our results showed that elevated FBXL6 expression primarily initiates and promotes hepatocarcinogenesis and lung metastasis in mice. The effect of elevated FBXL6 expression was much stronger than that of Kras mutation (constitutive activation of Kras), p53 deletion, or Tsc1 loss on HCC metastasis in mice^41^.

To clarify the mechanism underlying FBXL6-driven HCC metastasis, we carried out comprehensive proteome-wide analysis of the FBXL6-regulated proteome, ubiquitinome, and interactome. The advantage of this study is that FBXL6-driven HCC tumors from Fbxl6 knock-in mice, which may more faithfully reproduce the pathogenesis of HCC initiation and development, were used instead of cell lines for comprehensive proteomics analysis. However, the method used in this study may have its own limitations. Current approaches based on proteomics or ubiquitomics analysis still cannot completely cover all cellular proteins. Therefore, low-abundance proteins may have been missed in our proteomics analysis. In addition, for ubiquitomics analysis, we used an anti-ubiquitin antibody to enrich tryptic peptides, but the enrichment of ubiquitinated peptides could be dependent on antibody specificity. In addition, our immunoprecipitation experiment likely did not detect the weak interaction partners of FBXL6. Due to these limitations, we considered whether a potential substrate of FBXL6 could be identified in two of the three independent datasets.

In this study, we observed that HSP90AA1 and p53 were not ubiquitinated in the tumors of Fbxl6 knock-in mice, indicating that HSP90AA1 and p53 may not be the key molecules responsible for FBXL6-driven liver tumorigenesis and lung metastasis in vivo. In addition, previously reported potential substrates of FBXL6, including CCNA2 and VDAC2, were not involved in the oncogenic role of FBXL6 in HCC, as indicated by the low binding affinity of these proteins for FBXL6 compared with that of TKT, suggesting that TKT is the predominant substrate of FBXL6. Our results reveal a mechanism of HCC metastasis that depends on the ubiquitination of TKT by FBXL6, highlighting the versatility of the FBXL6-TKT interaction. Whether TKT activity depends on its ubiquitination is not yet known. A previous study reported that AKT phosphorylated TKT and enhanced its activation^35^. Here, we found that VRK2 interacted with TKT and phosphorylated it at Thr287. This phosphorylated TKT was recognized and then ubiquitinated by the SCF-FBXL6 ubiquitin ligase for its activation. Activated TKT was localized in the cytoplasm, reduced ROS accumulation, activated mTOR, and induced HCC metastasis. Evidence has revealed that nuclear TKT promotes cardiomyocyte apoptosis^42^, supporting our finding that cytoplasmic TKT has robust biological activity. Thr287 phosphorylation of TKT and K63-linked ubiquitination of TKT at K16/319 were positively correlated with its cytoplasmic localization and activation.

VRK2 is abnormally upregulated in a series of cancers^43,44^, but the underlying mechanism is poorly understood. Interestingly, we found that mTOR increased VRK2 expression in cancer cells. Previous studies have reported that ROS can positively or negatively regulate mTOR activation^38^. Here, we found that inhibition of ROS significantly activated mTOR and then upregulated VRK2 expression, which further enhanced VRK2-mediated TKT activation. This finding further improved our current understanding of TKT activation. A positive correlation between the protein levels of activated TKT and VRK2 was observed in HCC patients. These findings delineate the mechanism underlying the activation of TKT and elevated VRK2 expression in cancers.

Immune checkpoint proteins, especially PD-L1, play a crucial role in helping cancer cells escape autoimmunity by reducing T-cell activity, thus promoting tumor growth and progression^45^. Here, we found that FBXL6-TKT pathway activity upregulated PD-L1 expression in hepatocytes via the ROS-mTOR axis and then promoted HCC tumorigenesis and lung metastasis. Tumors with PD-L1 expression may be therapeutic targets in cancers^46^. In line with this observation, we found that targeting TKT significantly reduced FBXL6-mediated PD-L1 expression and blocked HCC tumorigenesis and metastasis, suggesting TKT as a potential therapeutic target for patients with FBXL6-high HCC.

In conclusion, our data demonstrate that (1) FBXL6 is a biomarker of poor prognosis in a subset of patients with an aggressive subtype of HCC; (2) elevated FBXL6 expression in hepatocytes drives HCC metastasis in vivo; (3) FBXL6 facilitates K63-linked polyubiquitination and activation of TKT, leading to HCC tumorigenesis and metastasis; (4) TKT ubiquitination requires VRK2-mediated phosphorylation at Thr287; (5) activated TKT upregulates PD-L1 and VRK2 expression via the ROS-mTOR axis, leading to immune evasion and HCC metastasis; (6) inhibition of or interference with TKT is a potential therapeutic strategy for combating FBXL6-driven immune escape and HCC metastasis; and (7) the oncogenic activity of FBXL6 relies on activated TKT, which is a marker for an aggressive subtype of human HCC. Therefore, our findings provide a mechanistic basis for therapeutic efficacy in patients with an aggressive subtype of FBXL6-positive HCC by targeting TKT or interfering with its expression.

### Supplementary information

Supplementary information

## Data Availability

The data that support the findings of this study are available in the supplementary material of this article. The mass spectrometry-based proteomics data have been deposited into the ProteomeXchange Consortium (http://proteomecentral.proteomexchange.org) via the PRIDE partner repository under the dataset identifier PXD032357. The datasets and materials used or analyzed during the current study are available from the corresponding author on reasonable request.
