# Author Correction: Elevated FBXL6 expression in hepatocytes activates VRK2-transketolase-ROS-mTOR-mediated immune evasion and liver cancer metastasis in mice

**DOI:** 10.1038/s12276-023-01116-8

**Published:** 2023-10-02

**Authors:** Jie Zhang, Xiao-Tong Lin, Hong-Qiang Yu, Lei Fang, Di Wu, Yuan-Deng Luo, Yu-Jun Zhang, Chuan-Ming Xie

**Affiliations:** Key Laboratory of Hepatobiliary and Pancreatic Surgery, Institute of Hepatobiliary Surgery, Southwest Hospital, Third Military Medical University (Army Medical University), Chongqing, 400038 China

**Keywords:** Liver cancer, Ubiquitylation, Phosphorylation, Ubiquitylation, Protein-protein interaction networks

Correction to: *Experimental & Molecular Medicine* 10.1038/s12276-023-01060-7, published online 01 September 2023

After online publication of this article, the authors noticed several errors in the “Results” section.

The correct statement of this article should have read as below.

In Fig. 1d, authors realize some inadvertent errors during image assembly, in which the upper panel image of Tsc1^fl/fl^; Alb-Cre mice is misused from another repeated experiment with similar result and the same conclusion but not from the 13 mice used in this study. In addition, the upper panel images of Fbxl6^LSL-fl/+^;Alb-Cre in Fig. 1d and Fig. S4a are erroneously copied. We sincerely apologize for this and now we would like to provide a new and more representative Fig. 1d as following, in which the Fig. 1d has been replaced. The correction of Fig. 1d does not influence the conclusion of the in vivo functionality experiments or the overall conclusion of the paper.
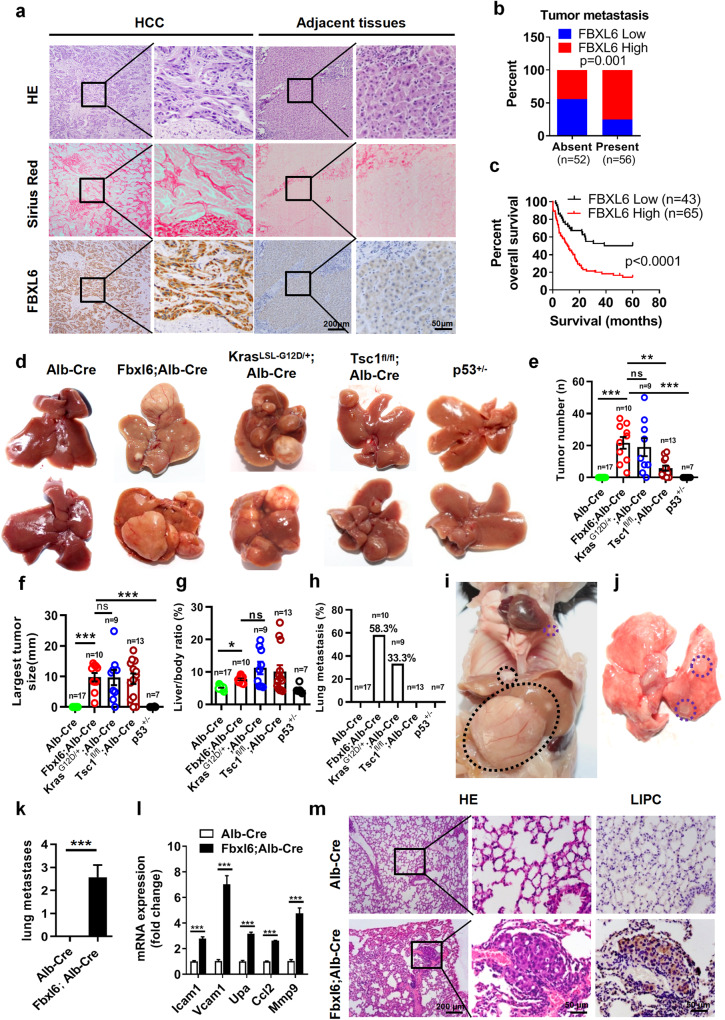


In Fig. 2f, the authors regret that there are mistakes in labeling sequence of TKT peptide. “13-QALKDTA-21” is replaced with “13-QALKDTA-19” and “45-QALKDTA-53” is replaced with “45-QALKDTA-51”. The updated figure is provided below. This does not impact the analysis, results, or conclusions of the article.
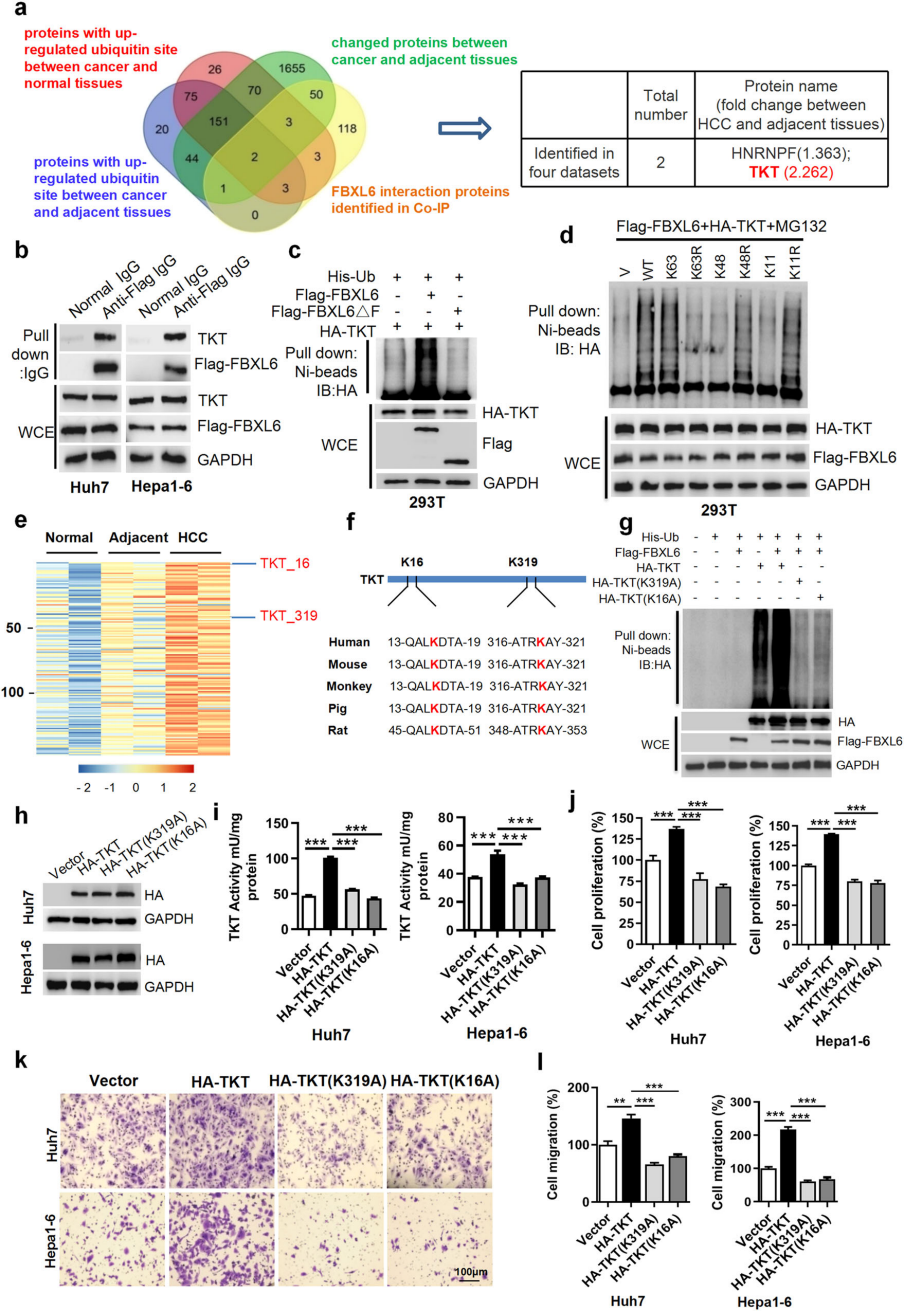


In Fig. 3a, the authors regret the labeling mistakes in sequence of TKT peptide. For Rat, “282-KKILVTPPQED-392”is changed to “314-KKILATPPQED-324.” For other species, “282-KKILATPPQED-392” is replaced with “282-KKILATPPQED-292”. The updated figure is provided below and do not impact the analysis, results, or conclusions of the article.
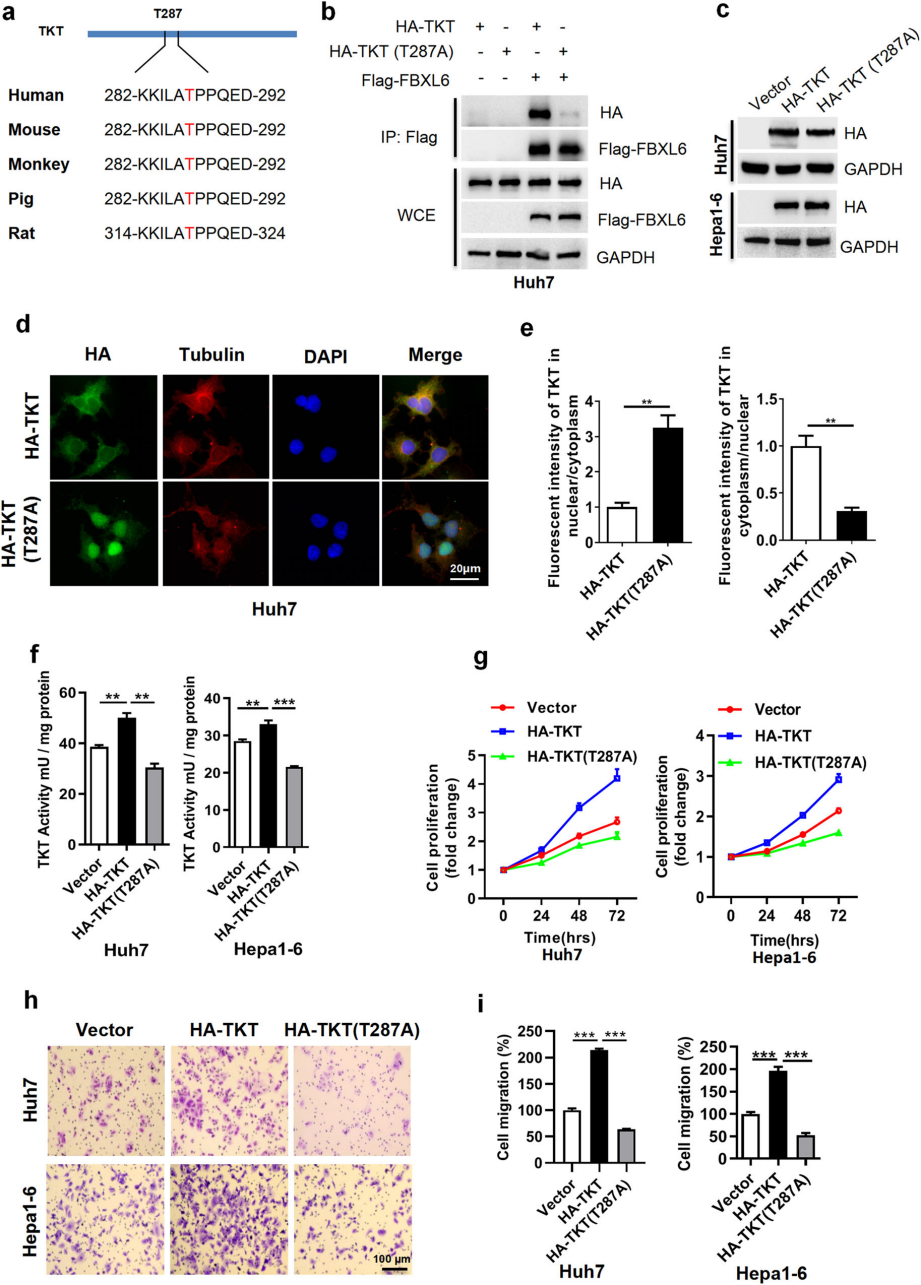


In addition, authors realize that Fig. 5c had been assembled incorrectly, in which the mTOR band in Hepa1-6 cells is a longer exposure time mTOR band of Huh7. The correct mTOR band for Hepa1-6 has been updated and shown below. This correction does not influence the conclusion of the paper.
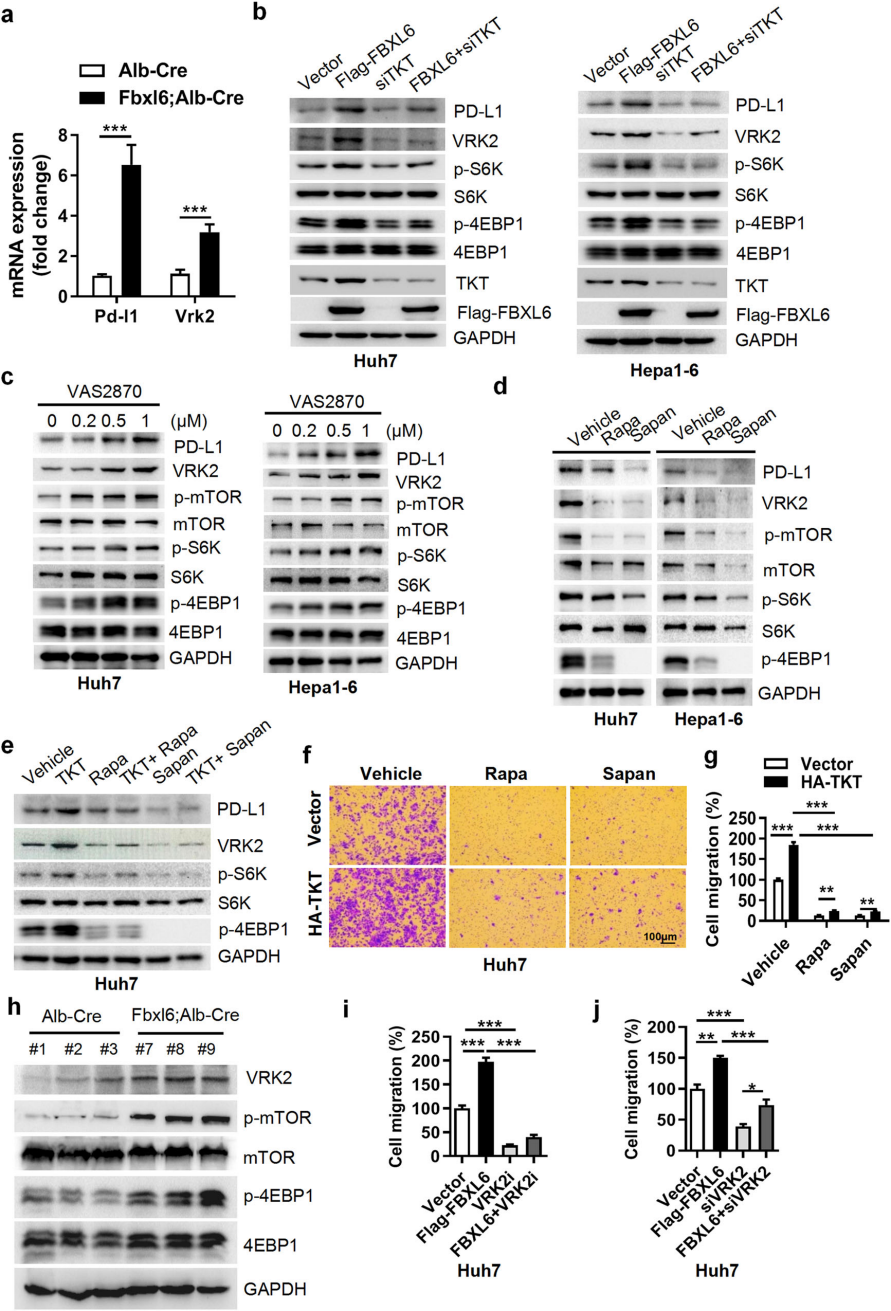


These corrections do not alter the conclusions of this article. The authors apologize for any inconvenience caused.

The original article has been corrected.

