# Supplementary material for: Elevated FBXL6 expression in hepatocytes activates VRK2-transketolase-ROS-mTOR-mediated immune evasion and liver cancer metastasis in mice
Source: Exp Mol Med. 2023 Sep 1;55(10):2162–76. doi: 10.1038/s12276-023-01060-7 (PMC10618235; doi:10.1038/s12276-023-01060-7)
Supplement: Supplementary file 1 — Supplementary information [file 12276_2023_1060_MOESM1_ESM.pdf]

Supplementary information

Elevated FBXL6 expression in hepatocytes activates VRK2-transketolase-ROS-mTOR-mediated immune evasion and liver cancer metastasis in mice

Supplementary Materials and Methods

Constructs

Human and mouse FBXL6 and TKT genes were synthesized and subsequently cloned into the pCDNA3.1 expression vector with a 3xFlag or 3xHA tag for ectopic expression.

Isolation of primary hepatocyte cells

The liver and portal tracts were exposed via a ventral midline incision, and a 22 G intravenous catheter was inserted into the portal vein. The liver was perfused at 7 mL/min via the portal vein for 10 min with pre-perfusion buffer (Gey's balanced salt solution (GBSS) without Ca^{2+} (NaCl: 7.0 g, KCl: 0.37 g, $MgSO_4 \cdot 7H_2O$: 70 mg, $Na_2HPO_4 \cdot 12H_2O$: 302 mg, $NaHCO_3$: 2.27 g, KH_2PO4: 30 mg, $MgCl_2 \cdot 6H_2O$: 210 mg, glucose: 1.0 g, EDTA: 0.744 g, pH 7.4)) at 37°C until the liver was completely discoloured and for 10 min with perfusion buffer (GBSS (NaCl: 7.0 g, KCl: 0.37 g, $MgSO_4 \cdot 7H_2O$: 70 mg, $Na_2HPO_4 \cdot 12H_2O$: 302 mg, $CaCl_2$: 166 mg, $NaHCO_3$: 2.27 g,

KH_2PO_4: 30 mg, $MgCl_2 \cdot 6H_2O$: 210 mg, glucose: 1.0 g, pH 7.4)) containing 0.16 mg/mL collagenase IV. After the two-step collagenase perfusion, the liver was excised, finely smashed by forceps in perfusion buffer, placed in a flask containing 0.16 mg/mL collagenase IV and 10 mg/mL DNase I, and placed in a shaking water bath at 37°C for 10 min. The resulting cell suspension was sequentially passed though a layer of 150-mesh nylon gauze to remove undigested tissue and a 70-mm cell strainer. The resulting single-cell suspension was obtained by pooling the filtered suspensions. The pellet was resuspended in PBS with 0.1% BSA and washed thee times to remove debris and other contaminants.

Cell lines, proliferation and cell migration assays

Mouse hepatocellular carcinoma Hepa1-6 cells were purchased from ATCC. Human Huh7 cells were purchased from the Cell Bank of Type Culture Collection of Fudan University (Shanghai, China). All cell lines tested negative for mycoplasma contamination. All experiments were carried out in DMEM medium containing 10% FBS, 100 U/mL penicillin and 100 mg/mL streptomycin (Invitrogen, USA) at 37°C and 5% CO_2.

For cell proliferation, Hepa1-6 and Huh7 cells were transfected with the indicated plasmids or siRNA oligos for 24 or 48 h using Lipofectamine 3000 transfection reagent. Cells were then seeded into 96-well plates at 2000 cells per well, and cell proliferation was analyzed using the Cell Counting Kit-8 (Dojindo, Japan) assay. For cell migration, $5x10^4$ cells were seeded into Transwell plates overnight,

stained with crystal violet (0.5% w/v) and imaged.

Small interfering RNA (siRNA) transfection

The siRNA oligos were purchased from GenePharma Co. (Shanghai, China) as follows: human siTKT #1, 5'-CUGCCGAACUGCUGAAGAA-3'; human siTKT #2, 5'-CAGGAGAUCUACAGCCAGA-3'; mouse siTKT #1, 5'-CCGUGGACAUUGCUAACAU-3'; mouse siTKT #2, 5'-GGAAUGUAUGACCACAGAUUU-3'; human siHNRNPF #1, 5'-GGAAGAAAUUGUUCAGUUC-3'; human siHNRNPF #2, 5'-GGUAUAUUGAAGUGUUUAAGA-3'; mouse siHNRNPF #1, 5'-GGUGUUCAUUUCAUUUAUACU-3'; mouse siHNRNPF #2, 5'-CCAAGCGAUGGAGUUGCAA-3'; human siFBXL6 #1, 5'-CACCGGCAUCAACCGUAAUAG-3'; human siFBXL6 #2, 5'-UGGAGUGGCUUAUGCCCAAUC-3'; mouse siFBXL6 #1, 5'-GCGAGAAGGACUUGGAACA-3'; mouse siFBXL6 #2, 5'-CGGCUGCUGAAUCUGAUUU-3'; non-targeting siRNA (siControl), 5'-UUCUCCGAACGUGUCACGUTT-3'; 5'- UCUACGAGGCACGAGACUU-3'. siRNA targeting VRK2 was purchased from Santa Cruz (sc-94622), and siJNK2 was purchased from Santa Cruz (sc-39101). Briefly, cells were transfected with various siRNAs in MEM medium with 90 nM of each siRNA duplex using Lipofectamine 3000 transfection reagent (Invitrogen) following the manufacturer's protocol.

RNA isolation and RT-qPCR and qPCR

Total RNA was isolated from cells or liver tissues with TRIzol (Life Technologies) or RNAiso Plus (TaKaRa), respectively. RNA was quantified using a NanoDrop spectrophotometer. Complementary DNA synthesis was carried out using the High-Capacity cDNA Reverse Transcription Kit (Applied Biosystems). Quantitative PCR (qPCR) was performed using Fast SYBR Green (Applied Biosystems) on a 7900HT Fast Real-time PCR system (Applied Biosystems). The primers used in this study were listed (Supplementary Table 6)

Western blotting

The treated cells were lysed in RIPA lysis buffer (Thermo Scientific, USA) containing a protease inhibitor mixture (Roche, Switzerland) on ice for 20 min. After centrifugation at 13000 rpm for 15 min at 4°C, the supernatant was collected, and the protein concentration was analyzed using a BCA protein assay reagent kit (Beyotime, China). Proteins were separated by SDS-PAGE (Beyotime, China) and then transferred to NC membranes (GE Healthcare, UK). Then, membranes were blocked with 5% nonfat milk and incubated with primary antibodies as follows: anti-mouse Flag (F1804, 1:2000, Sigma); anti-rat HA (12013819001, 1:2000, Roche); anti-rabbit FBXL6 (NBP1-31020, 1:1000, Novus Biologicals); anti-rabbit GAPDH (10494-1-AP, 1:5000, Proteintech); anti-rabbit VRK2 (DF2690, 1:1000, Affinity Bioscience); anti-rabbit PD-L1 (17952-1-AP, 1:1000, Proteintech); and anti-mouse transketolase (sc-390179, 1:1000, Santa Cruz Biotechnology) antibodies.

TKT polyubiquitination assay

To examine the ubiquitination of TKT by FBXL6, 293T cells were transfected His-Ub and Flag-FBXL6 with HA-TKT, HA-TKT (K319A), or HA-TKT (K16A). After 72 h of transfection, cells were treated with 10 μM MG132 for 4 h before harvest for the in vivo ubiquitination assay. The in vivo ubiquitination assay was performed as described previously using Ni-bead pull-down assays. In brief, the cells were lysed in buffer A (6 M guanidinium-HCl, 0.1 M Na_2HPO_4/NaH_2PO_4, 10 mM Tris-HCl (pH 8.0), 5 mM imidazole and 10 mM β-mercaptoethanol) on ice for 1 h, followed by exposure to 50 μL Ni-NTA beads (Qiagen, Valencia, CA) overnight. After incubation, beads were washed with buffer A plus 10 mM β-mercaptoethanol, buffer B (8 mM urea, 0.1 M Na_2HPO_4/NaH_2PO_4, 10 mM Tris/HCl, (pH 8.0), 10 mM β-mercaptoethanol), buffer C (8 mM urea, 0.1 M Na_2HPO_4/NaH2PO4, 10 mM Tris/HCl (pH 6.3), 10 mM β-mercaptoethanol, 0.2% Triton X-100), and buffer C plus 10 mM β-mercaptoethanol and 0.1% Triton X-100. His6-tagged ubiquitinated proteins were then eluted by buffer D (200 mM imidazole, 0.15 M Tris-HCl (pH 6.7), 30% glycerol, 0.72 M β-mercaptoethanol, 5% SDS), respectively. The eluate was analyzed using Western blotting.

Immunohistochemistry (IHC) staining assay

Briefly, tissues were fixed in 10% formalin and then embedded in paraffin. Sections of 5 μm were stained with H&E for histopathological examination or IHC staining with anti-rabbit FBXL6 (NBP1-31020, 1:100, Novus Biologicals),

anti-mouse transketolase (sc-390179, 1:100, Santa Cruz Biotechnology), anti-rabbit p-TKT(Th287) (generated by PTM Bio), anti-rabbit PD-L1 (#13684, 1:1000, Cell Signaling Technology); anti-rabbit CCNB2 (21644-1-AP, 1:200, Proteintech), anti-mouse MMP9 (10373-2-AP, 1:200, Proteintech), and anti-mouse Ki67 (550609, 1:100, BD Pharmingen) antibodies. The staining was evaluated by different specialized pathologists without any knowledge of the patient characteristics. The staining intensity was determined using the Spot Denso function of AlphaEaseFC software. The number of positively stained cells per 500 cells was recorded.

Co-immunoprecipitation (co-IP) assay

To examine the direct interaction between FBXL6 and TKT, Huh7, Hepa1-6, and HEK293T cells were transfected with Flag-FBXL6 alone or in combination with HA-TKT plasmids for 48 h, followed by lysis in immunoprecipitation lysate buffer (20 mM Tris-HCl, pH 8.0, 100 mM NaCl, 1% NP-40 with protease inhibitor) for 20 min on ice. Cell lysates were collected by centrifugation at 10000 g for 10 min at 4°C, and then 1-10 μL (0.2-2 μg) primary antibodies was added. After incubation for 1 h at 4°C, 50 μL Protein A/G PLUS-Agarose (Santa Cruz Biotechnology, USA) was added to the protein-antibody complex and incubated at 4°C overnight. Immunoprecipitates were washed thee times with immunoprecipitation buffer. A 2x sample loading buffer was added to the beads before boiling for 5 min. The supernatant was collected and used in the Western blotting assay.

In vitro ubiquitination assay

Flag-FBXL6 was precipitated from Flag-FBXL6 transfected HEK293 cells by protein A/G-beads(Santa), serving as the source of E3. HA-TKT and HA-TKT mutant (T287A) were precipitated from HA-TKT or HA-TKT mutant (T287A) transfected HEK293 cells, and eluted with 3xHA peptide (Beyotime), serving as the source of substrates. The reaction was carried out at 30°C for 2 h in 30 mL reaction buffer (40 mM Tris-HCl, pH 7.5, 2 mM DTT, 5 mM MgCl2) in the presence of Flag-FBXL6 (bound to protein A/G beads), ubiquitin (Boston Biochem), E1 (Boston Biochem), recombinant UbcH5c/E2 (Boston Biochem), ATP, and HA-purified TKT or its mutant T287A. After the reaction, the beads were washed thee times with cell lysis buffer to remove non-TKT conjugated ubiquitin. The washed beads were then resuspended in 25 μL 2xSDS-PAGE sample buffer for SDS-PAGE and detected by Western blotting with antibodies against HA.

ROS, NADPH and TKT enzymatic assays

For ROS assays, an equal number of cells were stained with 10 μM 2',7'-dichlorodihydrofluorescein diacetate (DCFHDA) (Life Technologies) and analyzed by BD FACSCanto II Analyzer. For the NADPH/NADP$^+$ ratio, NADPH and NADP$^+$ were measured by an NADPH/NADP$^+$ detection kit (Beyotime) according to the manufacturer's instructions. Briefly, HCC cells were lysed by NADP$^+$/NADPH extraction buffer (Beyotime). A portion of the samples was unheated, and another portion was heated at 60°C for 30 min for NADP decomposition. Heated and

unheated samples were incubated with NADPH developer at 37°C for 30 min in the dark and measured at OD450 using a plate reader. The NADPH/NADP$^+$ ratio was calculated by the following equation: (intensity of heated)/(intensity of unheated-heated samples). For TKT activity, cell lysates were harvested and added TKT reaction mix and immediately started recording fluorescence (Ex/Em = 535/587 nm) at 30 sec intervals for 30-45 min at 37°C using TKT activity kit (Biovision; # K2004-100) according to the manufacturer's instructions. Sample TKT Specific Activity=ΔM x D/ (Δt x P) (pmol / (min x µg)) = µUnits/µg or mUnits/mg. Where ΔM = G3P concentration from the Standard Curve (pmol); Δt = t2 – t1 (min); D = Sample dilution factor; and P = Sample used (in µg).

Proteomics, phosphoproteomics and ubiquitomics analysis

Liver tissues were added to four volumes of lysis buffer (8 M urea, 1% Protease Inhibitor Cocktail), followed by sonication thee times. The remaining debris was removed by centrifugation at 12,000 g at 4°C for 10 min. The protein concentration was determined with a BCA kit according to the manufacturer's instructions. For digestion, the protein was reduced with 5 mM dithiotheitol for 30 min at 56°C and alkylated with 11 mM iodoacetamide for 15 min at room temperature in darkness. Finally, trypsin was added overnight for digestion. For LC-MS/MS analysis, the tryptic peptides were dissolved in 0.1% formic acid (solvent A) and directly loaded onto a homemade reversed-phase analytical column (15-cm length, 75 µm i.d.). The gradient was comprised of an increase from 6% to 23% solvent B (0.1%

formic acid in 98% acetonitrile) over 26 min, 23% to 35% in 8 min and climbing to 80% in 3 min and then holding at 80% for the last 3 min, all at a constant flow rate of 400 nL/min on an EASY-nLC 1000 UPLC system. The peptides were subjected to NSI source followed by tandem mass spectrometry (MS/MS) in Q Exactive Plus TM (Thermo Scientific) coupled online to the UPLC. The electrospray voltage applied was 2.0 kV. The m/z scan range was 350 to 1800 for full scan, and intact peptides were detected in the Orbitrap at a resolution of 70,000. Peptides were then selected for MS/MS using the NCE setting of 28, and the fragments were detected in the Orbitrap at a resolution of 17,500. A data-dependent procedure that alternated between one MS scan followed by 20 MS/MS scans with 15.0 s dynamic exclusion. Automatic gain control (AGC) was set at 5E4. The fixed first mass was set as 100 m/z. The resulting MS/MS data were processed using the Maxquant search engine (v.1.5.2.8). *P*-values < 0.05 and fold changes of 1.2 (proteome data) and 1.5 (ubiquitome data) were considered significant.

Supplementary figure and legends

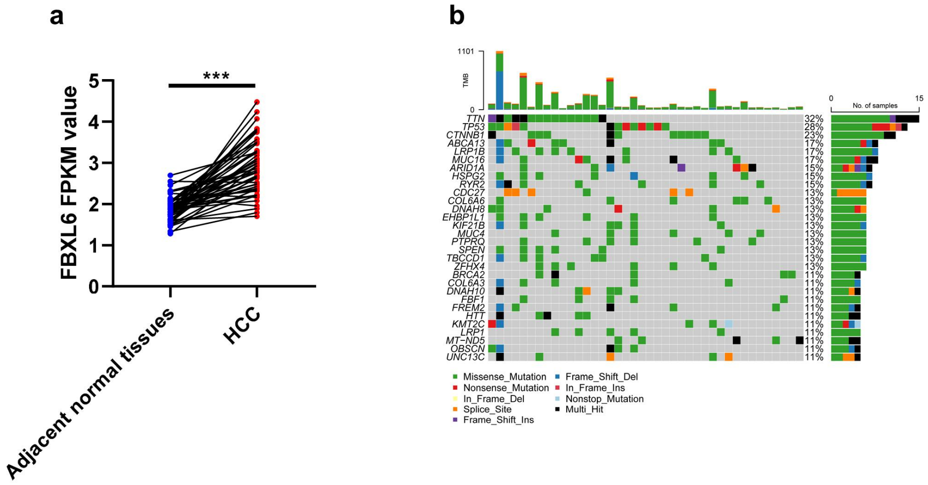

Fig. S1. Expression of FBXL6 in HCC and mutational profiles for the FBXL6 overexpressed HCC patients

a The FBXL6 mRNA level in 50 paired HCC and adjacent normal tissues was statistically analyzed in TCGA database. **b** The panel showing the mutational profiles for HCC patients with high FBXL6 expression. Two-tailed unpaired t-test was used in (**a**). ***$p \leq 0.001$.

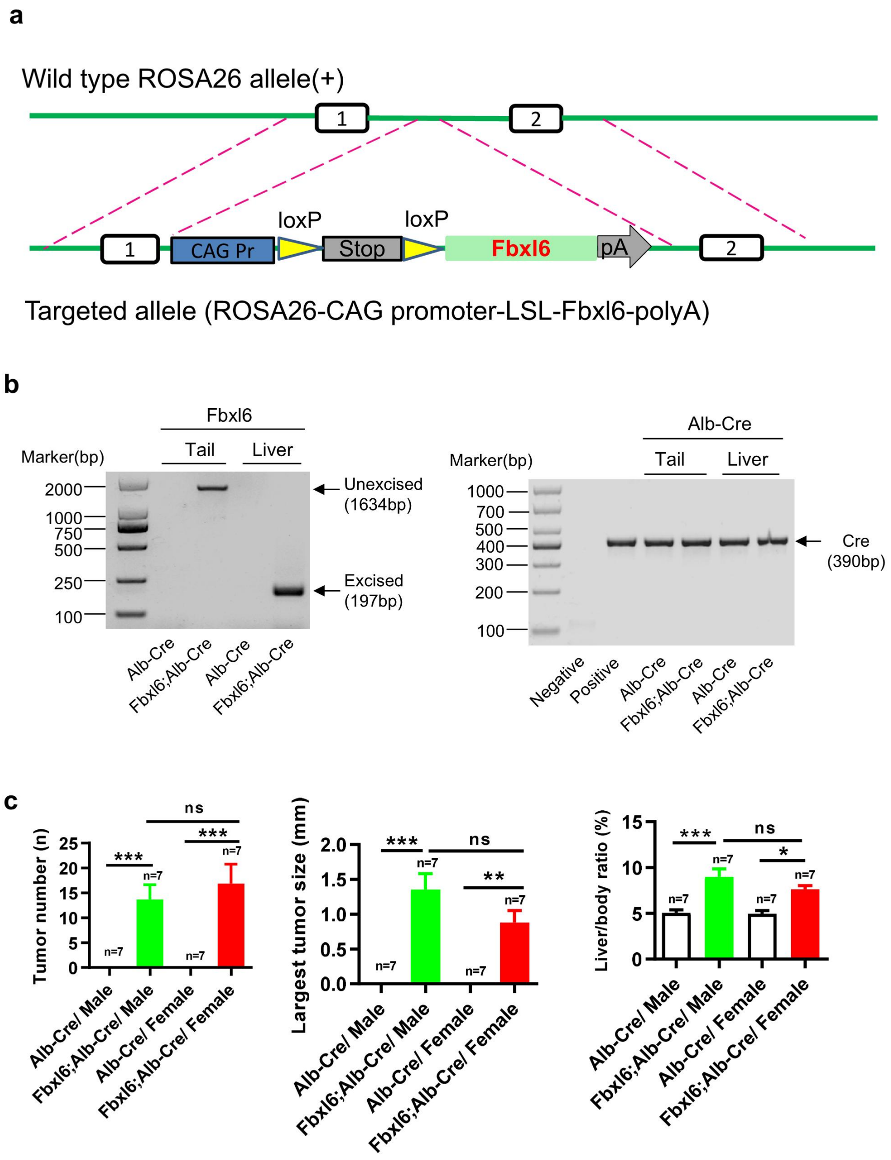

Fig. S2. FBXL6 promotes sex-independent hepatocarcinogenesis in vivo

a The scheme of the transgene construction of FBXL6 mice. **b** DNA was isolated from tails or livers of the indicated genotype and subjected to PCR genotyping for *Flox-Fbxl6*. **c** The tumor number, largest tumor size and liver/body weight ratio in 310-day female and male mice were compared. FBXL6 induced liver tumorigenesis independent of sex. Data are represented as the means ± SEMs. One-way ANOVA with Bonferroni's multiple comparisons test was used. n = 7 for each group. ns, nonsignificant. *$p \leq 0.05$, **$p \leq 0.01$, ***$p \leq 0.001$.

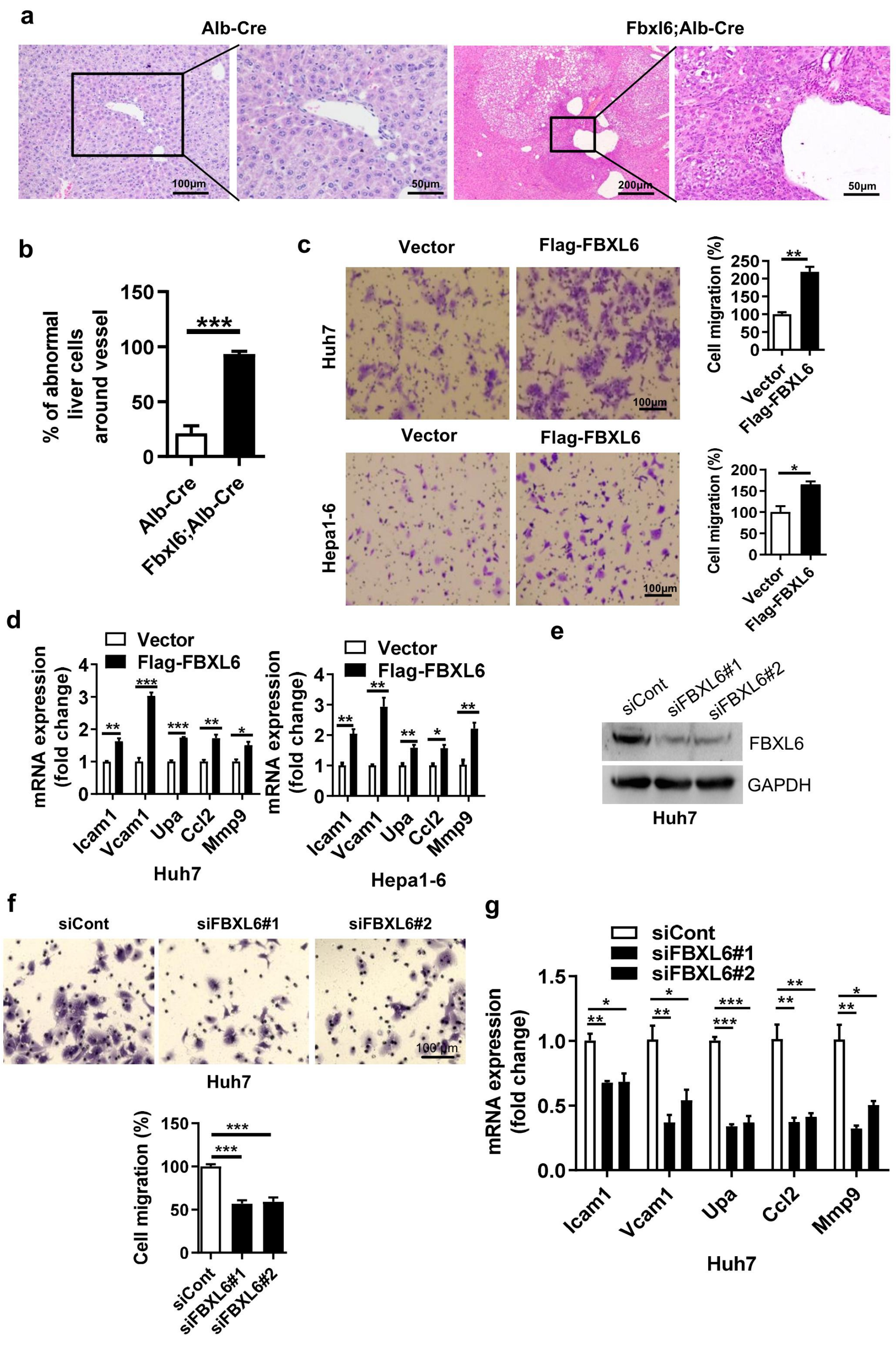

Fig. S3. FBXL6 promotes HCC cell migration.

a Representative images showing the HCC tumors around the blood vessels in *Fbxl6;Alb-Cre* mice. Scale bar, 200, 100, or 50 μm. **b** The abnormal cells around vessels were quantified (*n* = 8 mice for each group). **c**, **d** Liver cancer cells (Huh7 or Hepa1-6) were transfected with Flag-FBXL6 for 48 h and were then replated into

Transwell plates overnight (**c**). *n* = 4, biological replicates; migration markers (*Icam1, Vcam1, Upa, Ccl2, and Mmp9*) were analyzed by qPCR (**d**). *n* = 3, biological replicates. **e-g** Huh7 cells were transfected with siRNA targeting FBXL6 (siFBXL6) for 48 h. A portion of cells were used for Western blotting (**e**). Another portion was reseeded into Transwell plates for migration analysis after overnight incubation (**f**). The other portions were used to analyze metastasis-related markers (*Icam1*, *Vcam1*, *Upa*, *Ccl2*, and *Mmp9*) by qPCR (**g**). *n* = 3, biological replicates; Data are represented as the means ± SEMs. Scale bar, 100 μm. Two-tailed unpaired t-test was used in (**b**, **c**); One-way ANOVA with Tukey's multiple comparisons test was used in **(f);** Two-way ANOVA with Bonferroni's multiple comparisons test was used in (**d**, **g**). $*p \leq 0.05$, $**p \leq 0.01$, $***p \leq 0.001$.

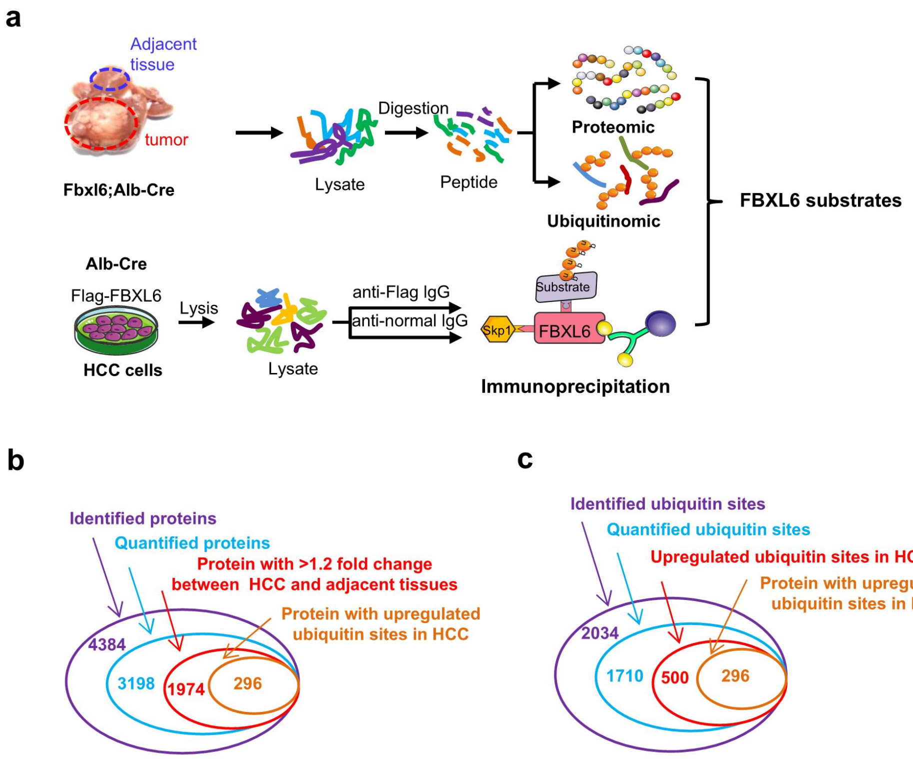

Fig. S4. Proteomics and ubiquitomics features of HCC tumors in response to FBXL6 overexpression

a Schematic of the multi-omics analyses of FBXL6-driven HCC tumors. The diagram shows the experimental process for the label-free proteomics, ubiquitomics, protein–protein interaction analysis of FBXL6. **b** The diagram showing the number of identified proteins (purple), quantified proteins (blue), proteins with significant changes (a cutoff of ≥ 1.2-fold change; red), and proteins with upregulated ubiquitin sites (a cutoff of ≥ 1.5-fold change; orange) in HCC tumors. **c** The diagram showing the number of identified ubiquitin sites (purple), quantified ubiquitin sites (blue), upregulated ubiquitin sites (a cutoff of ≥ 1.5-fold change; red), and proteins with upregulated ubiquitin sites (a cutoff of ≥ 1.2-fold change; orange) in HCC tumors.

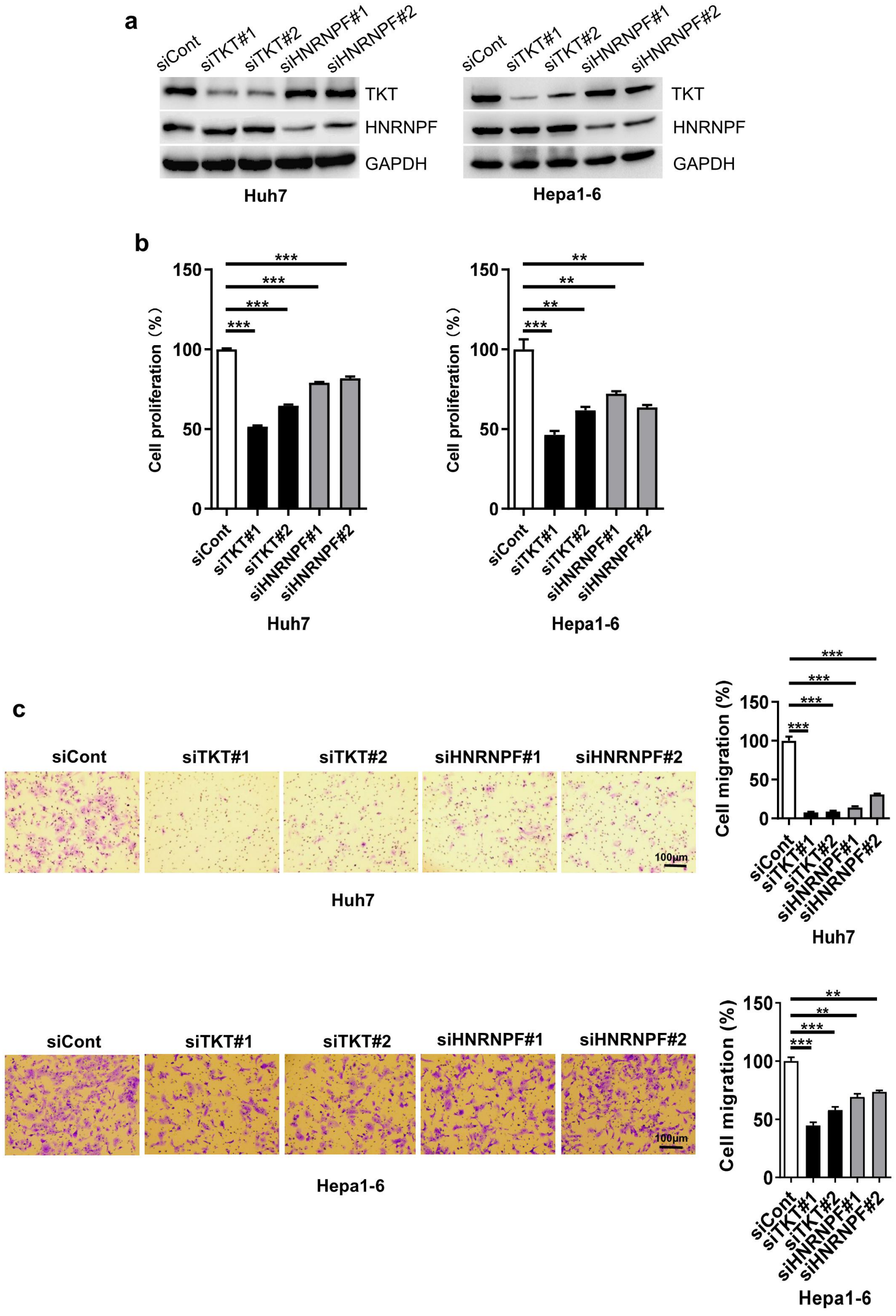

Fig. S5. TKT knockdown dramatically inhibits cell proliferation and migration in HCCs compared with HNRNPF knockdown

a-c Huh7 and Hepa1-6 cells were transfected with indicated siRNA oligos for 48 h, followed by Western blotting with indicated antibodies (**a**), cell proliferation analysis using CCK-8 assay (**b**), or cell migration assay (**c**). One-way ANOVA with Tukey's multiple comparisons test was used. $^{**}p \leq 0.01$, $^{***}p \leq 0.001$.

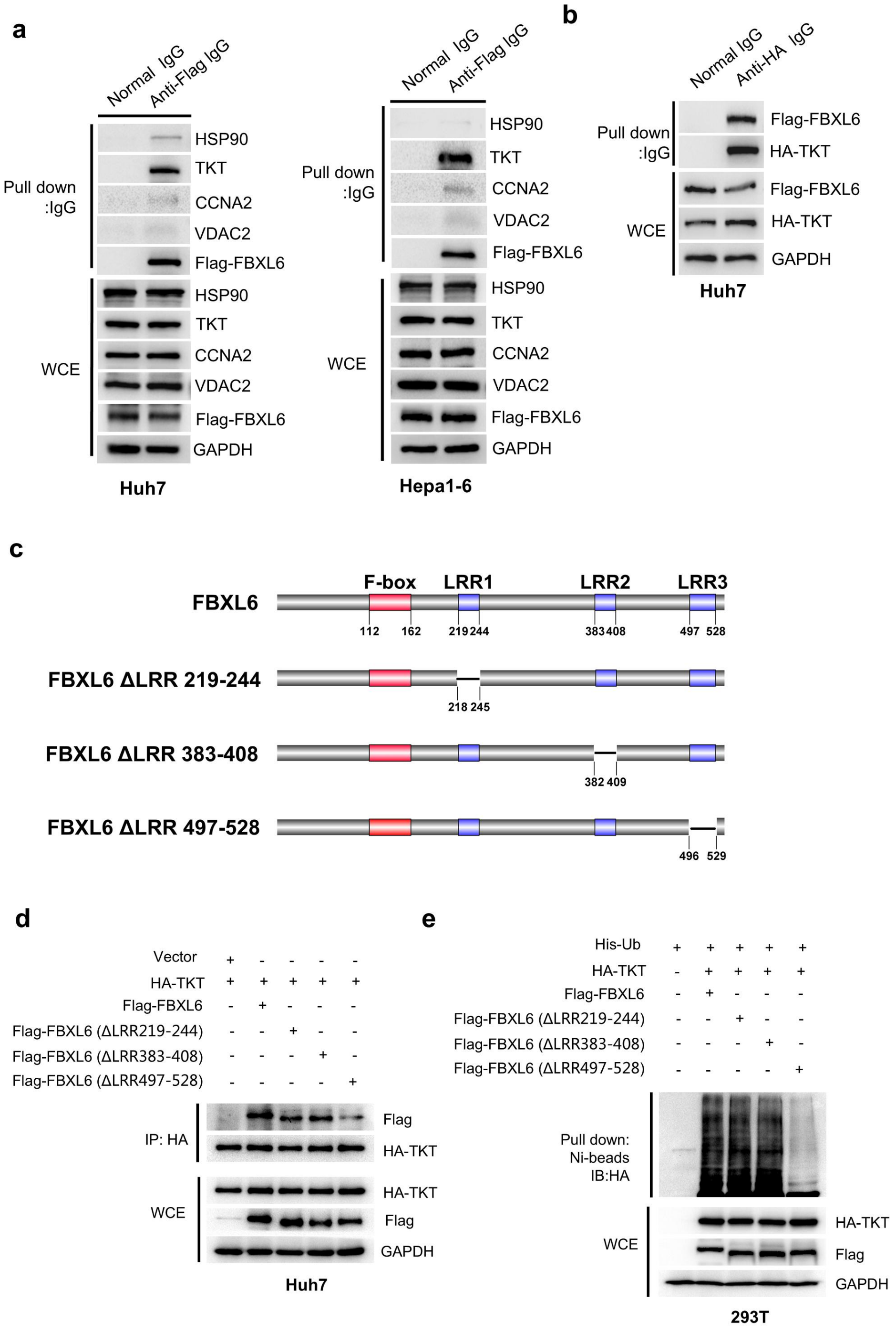

Fig. S6. FBXL6 LRR497-528 domain binds with TKT

a, **b** FBXL6 binds with TKT rather than other potential substrates in HCCs. Huh7 or Hepa1-6 cells were transfected with Flag-FBXL6 or HA-TKT plasmids for 45 h, followed by treatment with 3 h MG132 (10 μM) and lysis in RIPA buffer. Lysates

were added to the indicated antibodies and Protein A/G PLUS-Agarose. **c** Schematic structure of leucine-rich repeat (LRR) domain mutants in human FBXL6. **d** Huh7 cells were transfected HA-TKT with Flag-FBXL6 or its mutants for 48 h and then followed by co-IP with anti-HA antibody. **e** HEK293T cells were transfected HA-TKT and his-Ub with Flag-FBXL6 or its mutants for 45 h and then treated with 3 h MG132 (10 μM) . Cells were lysed in RIPA buffer, followed by pull-down with Ni-NTA beads or direct Western blotting with the indicated antibodies.

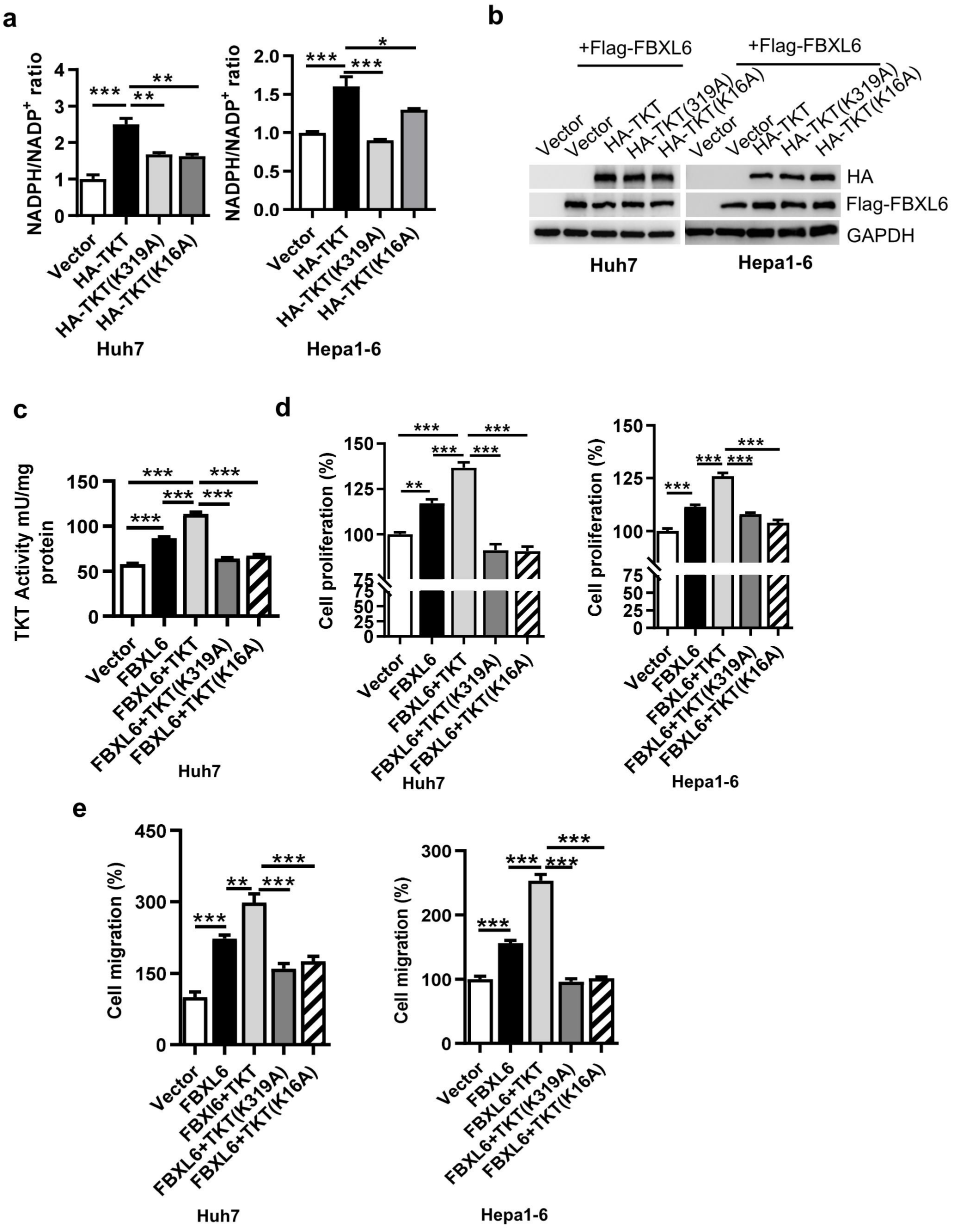

Fig. S7. K16 and K319 mutants in TKT attenuate TKT activity and inhibit cell migration

a Huh7 or Hepa1-6 cells were transfected with the indicated plasmids for 48 h. The NADPH/NADP$^+$ ratio was measured using an NADPH/NADP$^+$ detection kit. n = 3, biological replicates. **b-e** Huh7 or Hepa1-6 cells were transfected Flag-FBXL6 with HA-TKT, HA-TKT (K319A), or HA-TKT (K16A) for 48 h. A portion of the cells

were harvested for Western blotting (**b**) or detection of TKT activity using a TKT specific activity kit (**c**). Another portion was used for cell proliferation analysis (**d**). Other portions were reseeded into Transwell plates for migration analysis (**e**). One-way ANOVA with Tukey's multiple comparisons test was used. n = 3-4, biological replicates. $*p \le 0.05$, $**p \le 0.01$, $***p \le 0.001$.

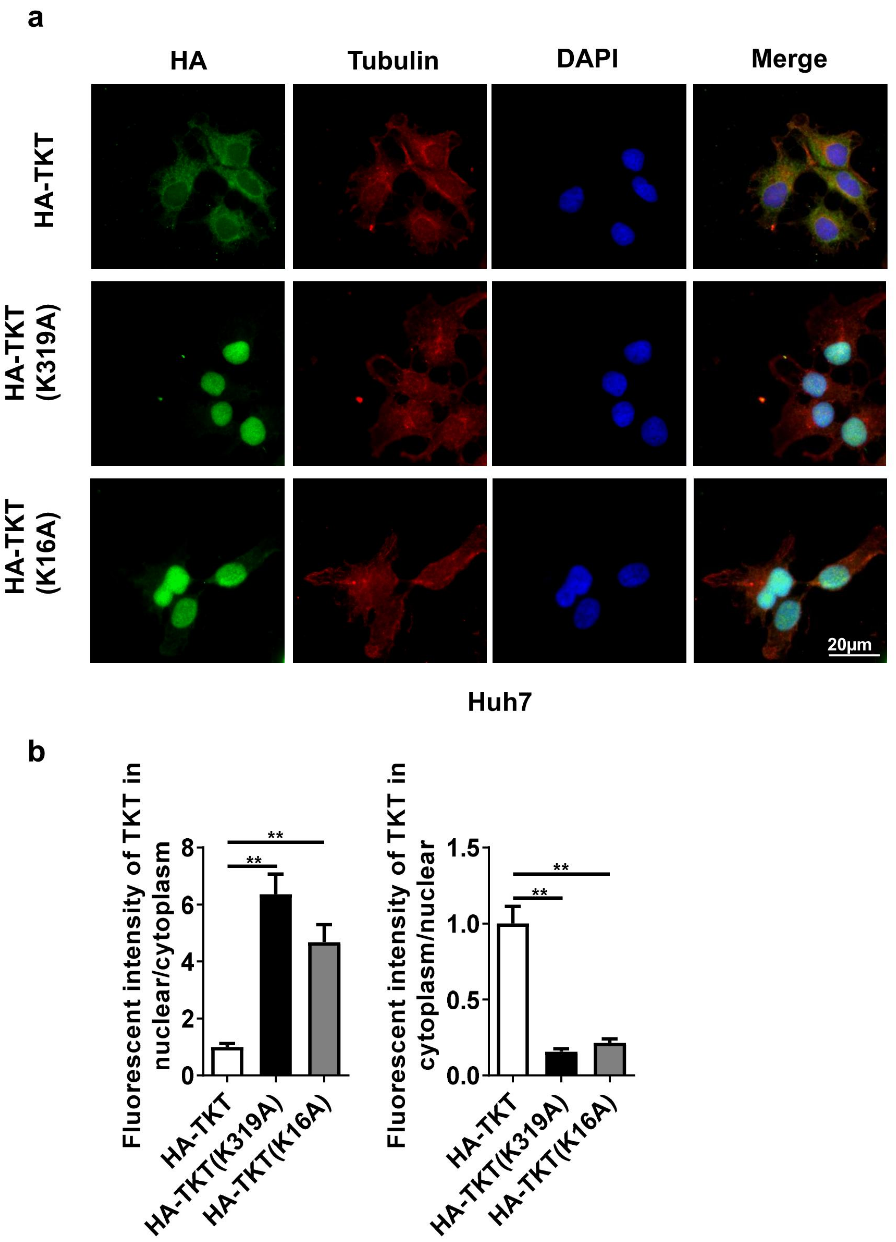

Fig. S8. K16 and K319 mutants in TKT promote its nuclear localization

a, **b** Huh7 cells were transfected with the indicated plasmids for 48 h and stained with anti-HA antibody. TKT wild-type localized at the cytoplasm, whereas its mutants (K16A and K319A) were in the nuclear (**a**). The nuclear and cytoplasmic fluorescence intensity in (**a**) was determined by ImageJ software (**b**). Scale bar, 20 μm. One-way ANOVA with Tukey's multiple comparisons test was used. $^{**}p \leq 0.01$.

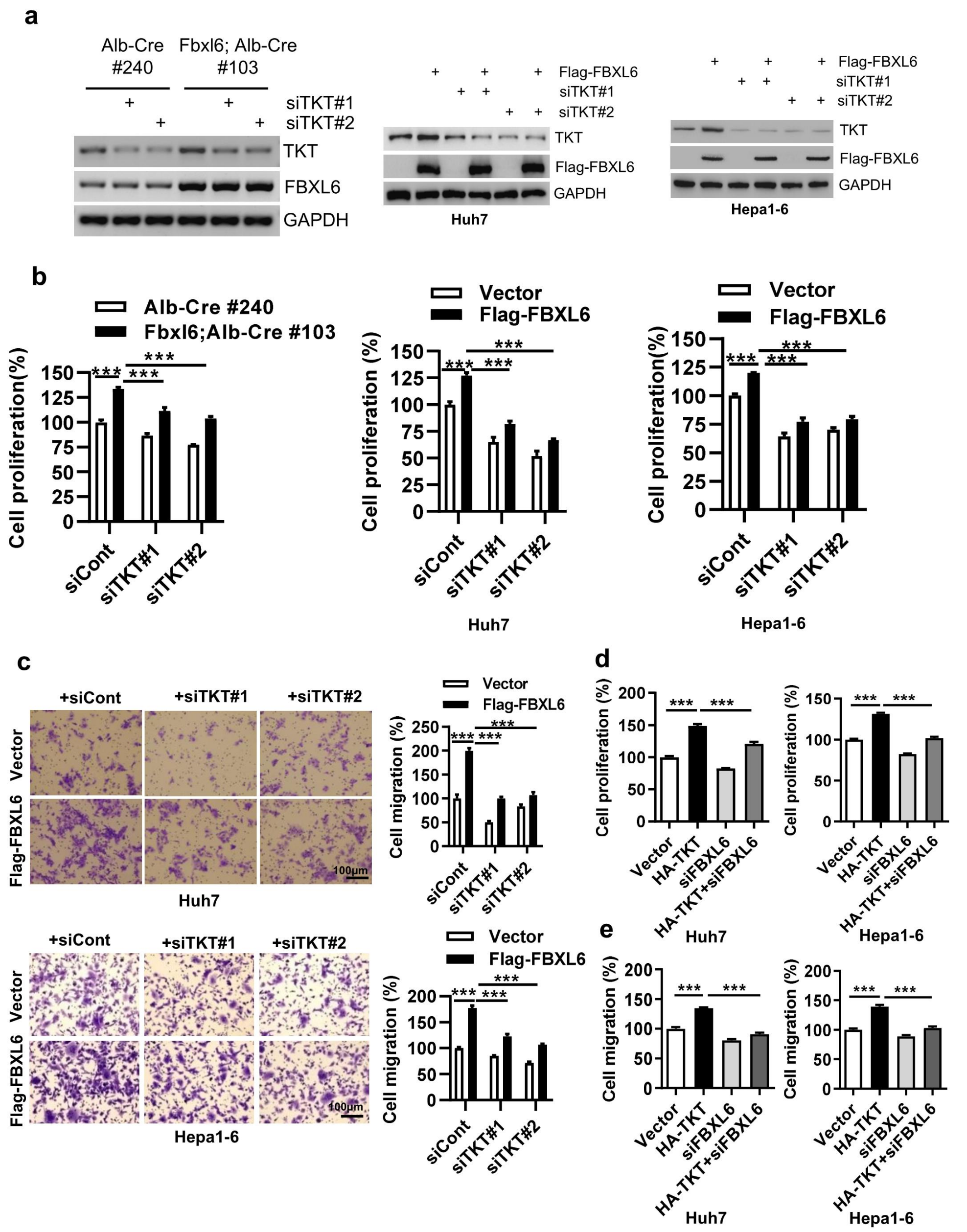

Fig. S9. TKT knockdown blocks FBXL6-mediated cell proliferation and migration in primary hepatocytes and HCCs

a-c Primary hepatocytes isolated from the age of 10 weeks *Alb-Cre* or *Fbxl6;Alb-Cre* mice, Huh7 and Hepa1-6 were transfected with indicated siRNA oligos for 48 h. A portion of cells was harvested for Western blotting (**a**). Another portion was plated into 96-well plates for 48 h and analyzed by CCK-8 assay (**b**). The other portions were seeded into Transwell plates for migration analysis (**c**). **d**, **e** Huh7 or Hepa1-6

cells were transfected HA-TKT with non-targeting siRNA control or siRNA targeting FBXL6 for 48 h. Another portion was plated into 96-well plates for 48 h and analyzed by CCK-8 assay (**d**). The other portions were seeded into Transwell plates for migration analysis (**e**). $n = 3$, biological replicates**.** Two-way ANOVA with Bonferroni post-tests was used in (**b**, **c**). One-way ANOVA with Tukey's multiple comparisons test was used (**d**, **e**). ***$p \leq 0.001$.

a

Protein accession	Protein description	Gene name	Position	Amino acid	HCC/ Adjacent tissue Ratio	Regulated Type	Localization probability	Modified sequence
P40142	Transketolase	Tkt	287	T	1.583	Up	1	ILAT(1)PPQEDAPS VDIANIR

b

site	code	kinase	peptide	scores	cut-off
4	T	CMGC/MAPK/JNK/JNK2	****ILATPPQEDAP	36.471	6.118
4	T	CK1/VRK/VRK2	****ILATPPQEDAP	25.25	4
4	T	CMGC/CDK/CDK5/CDK5	****ILATPPQEDAP	23.889	7.867
4	T	CMGC/MAPK/ERK/Erk1	****ILATPPQEDAP	23.25	10.326
4	T	CMGC/CDK/CDK2/CDK2	****ILATPPQEDAP	21.43	6.826
4	T	CMGC/CDK/CDK5	****ILATPPQEDAP	18.646	6.616
4	T	CMGC/MAPK/ERK/Erk2	****ILATPPQEDAP	13.867	4.61
4	T	STE/STE7/MEK7	****ILATPPQEDAP	12.833	7.32
4	T	CMGC/CDK/CDC2/CDK1	****ILATPPQEDAP	12.451	4.168
4	T	Atypical/PIKK/FRAP	****ILATPPQEDAP	12.417	8.145
4	T	CMGC/CDK/CDK2/CDC28	****ILATPPQEDAP	12.056	9.411
4	T	CMGC/MAPK/p38	****ILATPPQEDAP	11.83	4.309
4	T	STE/STE7/MEK7/MAP2K7	****ILATPPQEDAP	11.167	6.435
4	T	CMGC/MAPK	****ILATPPQEDAP	10.883	4.55
4	T	CAMK/CAMKL/QIK/SIK1	****ILATPPQEDAP	10.188	2.597

Fig. S10. Th287 in TKT is phosphorylated in FBXL6-overexpressed tumors

a Th287 in TKT was phosphorylated in HCC tumors of FBXL6 hyperexpression (*Fbxl6; Alb-Cre*) mice. **b** Predicted kinases for TKT phosphorylation at the Th287 site based on GPS2.0 software.

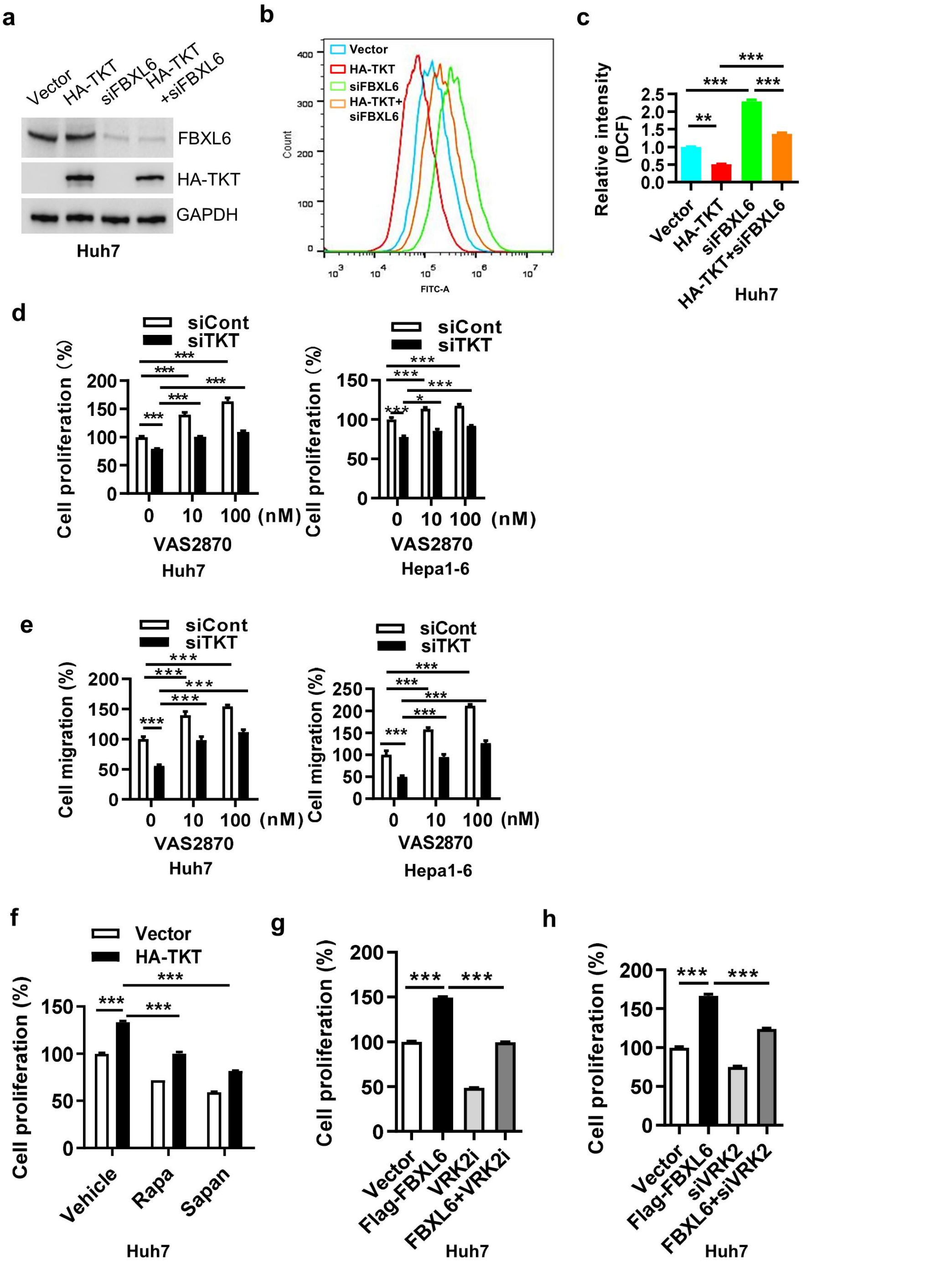

Fig. S11. TKT-ROS-mTOR-VRK2 axis plays a critical role in FBXL6-mediated HCC proliferation

a-c Huh7 cells were transfected HA-TKT with or without siRNA targeting FBXL6 for

48 h and then analyzed by Western blotting with indicated antibodies (**a**) or ROS analysis after DCFHDA staining (**b**, **c**). **d**, **e** Inhibition of ROS rescues TKT knockdown-induced the inhibition of cell proliferation and migration. Huh7 or Hepa1-6 cells were transfected with the indicated siRNA oligos for 24 h. A portion of cells were plated into 96-well plate and then exposed to the ROS inhibitor VAS2870 for 48 h. Cell proliferation was analyzed by CCK-8 assay (**d**). The other portions were plated into Transwell plates overnight with the ROS inhibitor VAS2870, and migration was quantified after crystal violet (0.5% w/v) staining (**e**). **f-h** Huh7 cells were transfected with indicated plasmids or siRNAs in the presence or absence of mTOR inhibitors (Rapamycine 100 nM; Sapanisertib 0.5 μM) or VRK2 inhibitor (IC261, 5 μM) for 48 h, and then stained with CCK-8 for cell proliferation analysis. Two-way ANOVA with Bonferroni's multiple comparisons test was used in (**d-f**); One-way ANOVA with Tukey's multiple comparisons test was used in (**c**, **g**, **h**). $^{*}p \leq 0.05$, $^{**}p \leq 0.01$, $^{***}p \leq 0.001$.

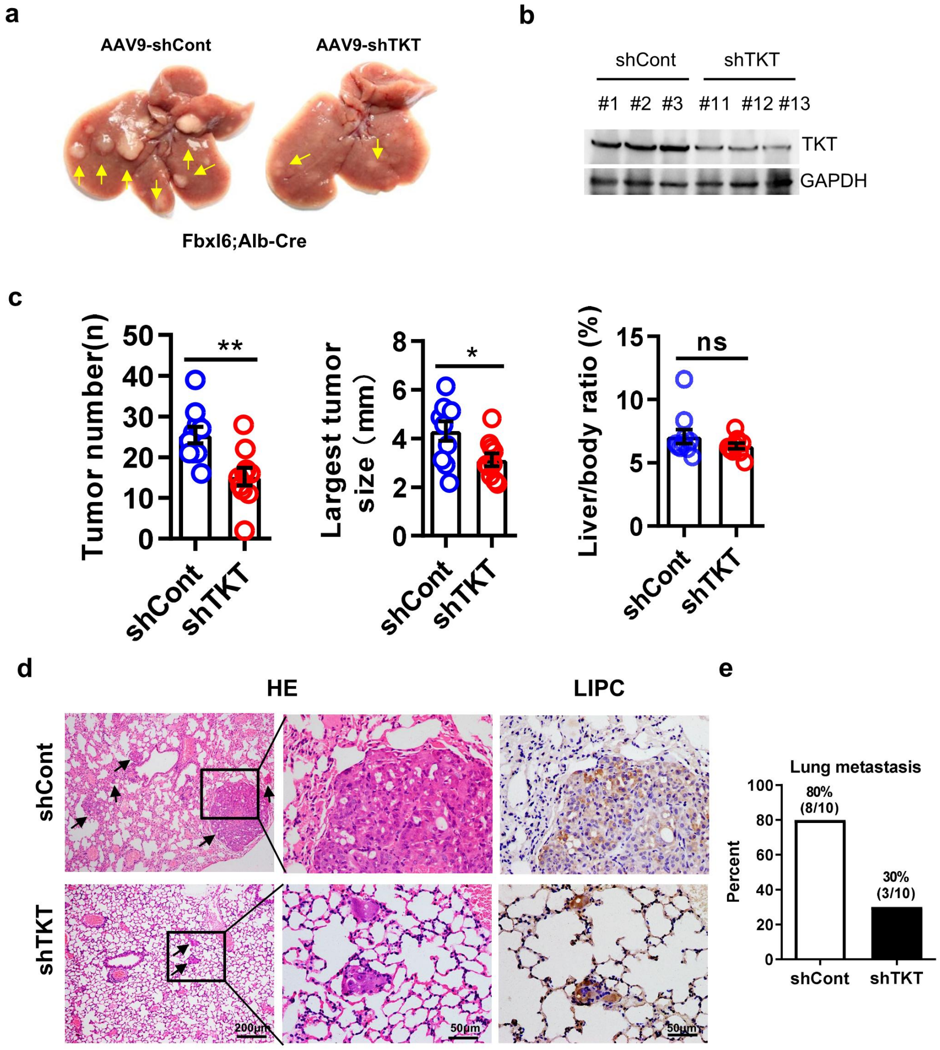

Fig. S12. Knockdown of TKT protects against FBXL6-triggered hepatocarcinogenesis in vivo

a-c *Fbxl6;Alb-Cre* (*n* = 20) mice were injected with DEN (25 mg/kg, i.p.) at the age of 5 weeks and subsequently administered 28 injections of CCl_4 (0.5 mL/kg) alone or a combination of adeno-associated virus AAV9-shTKT-eGFP-TBG ($5x10^{10}$; liver-restricted expression) after 4 injections of CCl_4. Mice were sacrificed 19 weeks after DEN. Shown are representative images (**a**), the efficiency of AAV9-shTKT (*n* = 3 mice per group) (**b**), tumor number, largest tumor size and liver/body weight ratio (**c**). **d** Representative H&E and IHC staining images showing the low rate of distinct lung metastatic foci expressing the hepatocytic marker lipase C after TKT knockdown.

Scale bar, 200 μm or 50 μm. **e** The lung metastasis rate was lower in the TKT knockdown group (30%, 3/10) than in the control group (80%, 8/10). $n = 10$ mice per group. Arrows indicate tumors. Two-tailed unpaired Student's t-test was used. ns: nonsignificant. $^{*}p \le 0.05$, $^{**}p \le 0.01$.

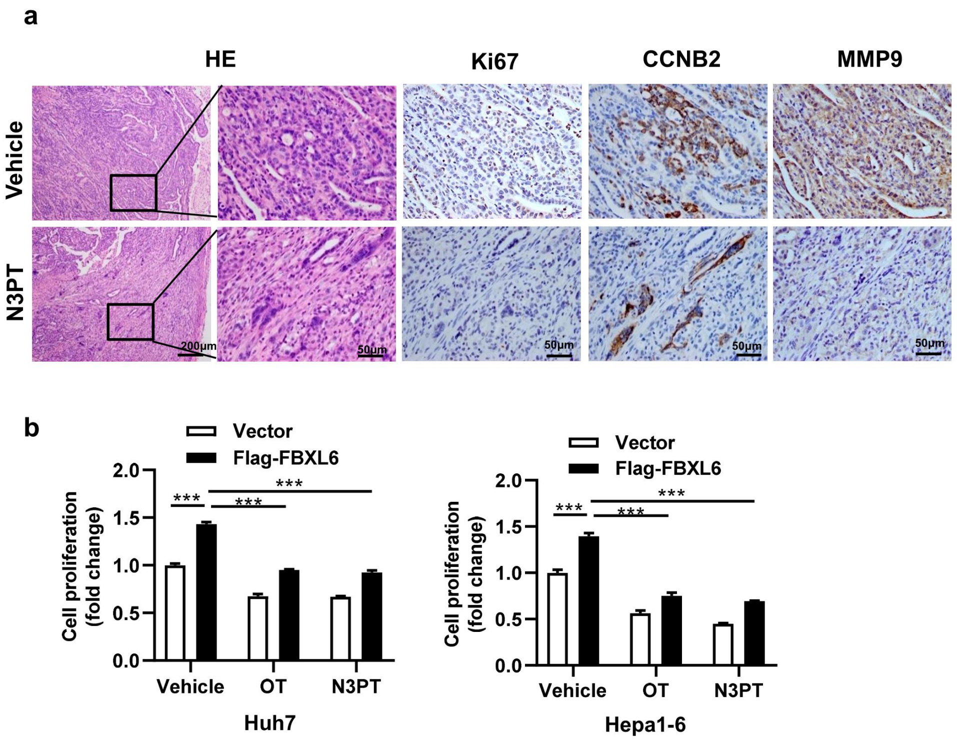

Fig. S13. Inhibition of TKT reduces proliferation- and metastasis-related markers

a Representative images showing the expression levels of Ki67, CCNB2 and MMP9 in liver tissues of vehicle- and N3PT-treated mice. **b** Huh7 or Hepa1-6 cells were transfected with Flag-FBXL6 for 24 h and then exposed to TKT inhibitors N3PT (0.5 μM) or OT (5 mM) for 48 h. Cell proliferation was analyzed by CCK-8 assay. Two-way ANOVA with Bonferroni's multiple comparisons test was used. ***$p \leq 0.001$.

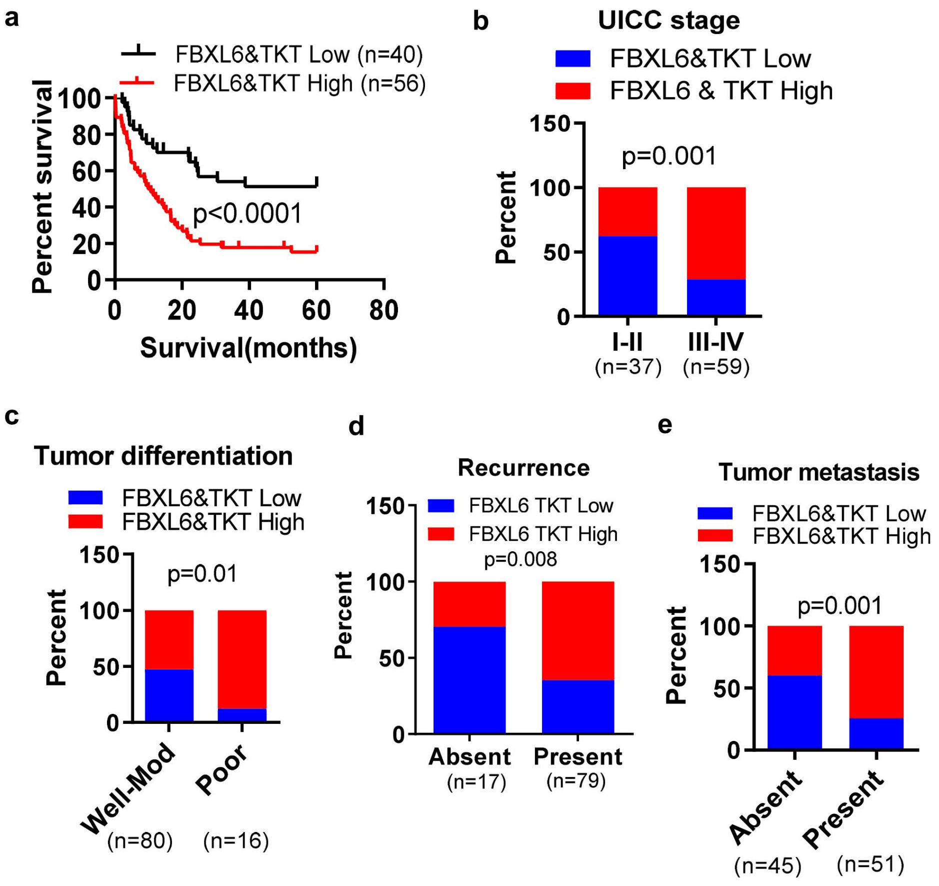

Fig. S14. Levels of FBXL6 and TKT are positively associated with poor clinical outcomes in HCC patients

a The association between high coexpression of FBXL6/TKT and overall survival in HCC patients was evaluated. The log-rank (Mantel-Cox) test was used. $p < 0.0001$. **b-e** The relationships between high coexpression of FBXL6/TKT protein and UICC stage (**b**), tumor differentiation (**c**), recurrence (**d**), and tumor metastasis (**e**) were determined.

Supplementary Table 1. Relationship between FBXL6 and clinicopathologic characteristics in the 108 HCC patients of the IHC cohort.

Variables	Cases(%)	Low FBXL6	High FBXL6	P Value
Age(years)				0.223
≤55	82(75.9%)	30	52	
>55	26(24.1%)	13	13	
Sex				0.249
Female	15(13.9%)	8	7	
Male	93(86.1%)	35	58	
Tumor Stage				**0.01**
T1/T2	59(54.6%)	30	29	
T3/T4	49(45.4%)	13	36	
Tumor size				**0.01**
≤5cm	24(22.2%)	15	9	
>5cm	84(77.8%)	28	56	
UICC Stage				**0.002**
I-II	43(39.8%)	25	18	
III-IV	65(60.2%)	18	47	
Histologic Grade				**0.032**
G1G2	92(85.2%)	41	51	
G3	16(14.8%)	2	14	
Vascular Thombosis				**0.013**
Absent	73(67.6%)	35	38	
Present	35(32.4%)	8	27	
Recurrence				**0.022**
Absent	19(17.6%)	12	7	
Present	89(82.4%)	31	58	
Metastasis				**0.001**
Absent	52(48.1%)	29	23	
Present	56(51.9%)	14	42	

***p* < 0.05 was considered for statistic significance, and was marked in bold.**

Supplementary Table 2. Univariate and multivariate analyses indicating associations between overall survival and various risk factors in the 108 HCC patients of the IHC cohort

Variables	N	OS	
		Hazard ratio(95% Cl)#	P value
**Univariate analysis**			
FBXL6(high vs. low)	(65 vs. 43)	2.701(1.625-4.489)	**<0.001**
Age(>55y vs. ≤55y)	(26 vs. 82)	0.772(0.450-1.324)	0.347
Gender(male vs. female)	(93 vs. 15)	1.622(0.780-3.377)	0.196
Histologic grade(G1G2 vs. G3)	(92 vs. 16)	2.170(1.221-3.856)	**0.008**
UICC Stage(I II vs. III IV)	(43 vs. 65)	4.151(2.409-7.155)	**<0.001**
Tumor Stage(T1 T2 vs. T3 T4)	(59 vs. 49)	3.693(2.281-5.98)	**<0.001**
Tumor size(>5cm vs. <=5cm)	(84 vs. 24)	3.017(1.546-5.886)	**0.001**
Recurrence(Present vs. Absent)	(89 vs. 19)	10.111(3.162-32.33)	**<0.001**
Vascular thombosis(Present vs. Absent)	(35 vs. 73)	3.263(2.033-5.236)	**<0.001**
Metastasis(Present vs. Absent)	(56 vs. 52)	3.136(1.929-5.099)	**<0.001**
**Multivariate analysis**			
FBXL6(high vs. low)	(65 vs. 43)	2(1.188-3.367)	**0.009**
Histologic grade (G1G2 vs. G3)	(92 vs. 16)	NA	NA
UICC Stage(I II vs. III IV)	(43 vs. 65)	NA	NA
Tumor Stage(T1 T2 vs. T3 T4)	(59 vs. 49)	2.364(1.442-3.875)	**0.001**
Tumor size(>5cm vs. <=5cm)	(84 vs. 24)	NA	NA
Recurrence(Present vs. Absent)	(89 vs. 19)	7.151(2.188-23.367)	**0.001**
Vascular thombosis(Present vs. Absent)	(35 vs. 73)	NA	NA
Metastasis(Present vs. Absent)	(56 vs. 52)	NA	NA

***p* < 0.05 was considered for statistic significance, and was marked in bold.**

Supplementary Table 3. Relationship between VRK2 and clinicopathologic characteristics in the 121 HCC patients of the IHC cohort.

Variables	Cases	Low VRK2	High VRK2	P Value
Age(years)	121	53	68	0.982
<55	89	39	50	
≥55	32	14	18	
Gender				0.578
Female	17	9	8	
Male	104	44	60	
TNM Stage				**0.001**
I - II	53	34	19	
III - IV	68	19	49	
Histologic Grade				0.358
G1G2	102	47	55	
G3	19	6	13	
Tumor Size				**0.003**
≤5cm	29	20	9	
>5cm	92	33	59	
Recurrence				**0.007**
Absent	34	22	12	
Present	87	31	56	
Vascular Thombosis				**0.018**
Absent	86	44	42	
Present	35	9	26	
Metastasis				**0.001**
Present	63	14	49	
Absent	58	39	19	

***p* < 0.05 was considered for statistic significance, and was marked in bold.**

Supplementary Table 4. Univariate and multivariate analyses indicating associations between overall survival and various risk factors in the 121 HCC patients of the IHC cohort

Variables	N	OS<br>Hazard ratio(95% Cl)#	P value
**Univariate analysis**			
VRK2(high vs. low)	(68 vs. 53)	0.3634(0.2314-0.5706)	**<0.001**
Age(>=55y vs. <55y)	(32 vs. 89)	0.8666(0.5396-1.392)	0.554
Gender(male vs. female)	(104 vs. 17)	1.324(0.7208-2.433)	0.365
Histologic grade(G1G2 vs. G3)	(102 vs. 19)	0.8444(0.4849-1.471)	0.55
TNM Stage(I II vs. III IV)	(53 vs. 68)	3.686(2.317-5.863)	**<0.001**
Tumor size(>5cm vs. <=5cm)	(92 vs. 29)	2.297(1.317-4.006)	**0.0034**
Recurrence(Present vs. Absent)	(87 vs. 34)	2.738(1.529-4.902)	**<0.001**
Vascular Thombosis(Present vs. Abs ent)	(35 vs. 86)	2.791(1.786-4.361)	**<0.001**
Metastasis(Present vs. Absent)	(63 vs. 58)	2.471(1.597-3.821)	**<0.001**
**Multivariate analysis**			
VRK2(high vs. low)	(68 vs. 53)	0.5744(0.3352-0.9841)	**0.0435**
TNM Stage(I II vs. III IV)	(53 vs. 68)	2.6768(1.4171-5.0564)	**0.0024**
Tumor size(>5cm vs. <=5cm)	(92 vs. 29)	1.0846(0.561-2.097)	0.809
Recurrence(Present vs. Absent)	(87 vs. 34)	1.9908(1.0551-3.7563)	**0.033**
Vascular Thombosis(Present vs. Abs ent)	(35 vs. 86)	1.3872(0.7861-2.4477)	0.258
Metastasis(Present vs. Absent)	(63 vs. 58)	0.7693(0.4105-1.4418)	0.413

***p* < 0.05 was considered for statistic significance, and was marked in bold.**

Supplementary Table 5. Relationship between activated p-TKT(T287) and clinicopathologic characteristics in the 121 HCC patients of the IHC cohort

Variables	Cases	Low p-TKT(T287)	High p-TKT (T287)	P Value
Age(years)	121	56	65	0.588
<55	89	43	46	
≥55	32	13	19	
Gender				0.74
Female	17	9	8	
Male	104	47	57	
TNM Stage				**0.001**
I - II	53	36	17	
III - IV	68	20	48	
Histologic Grade				0.883
G1G2	102	48	54	
G3	19	8	11	
Tumor Size				0.082
≤5cm	29	18	11	
>5cm	92	38	54	
Recurrence				**0.019**
Absent	34	22	12	
Present	87	34	53	
Vascular Thombosis				0.137
Absent	86	44	42	
Present	35	12	23	
Metastasis				**0.001**
Present	63	19	44	
Absent	58	37	21	

***p* < 0.05 was considered for statistic significance, and was marked in bold.**

Supplementary Table 6. Primers used for qRT-PCR

Gene symbol	Forward	Reverse
Mouse Icam1	CTGGGCTTGGAGACTCAGTG	CCACACTCTCCGGAAACGAA
Mouse Vcam1	CTGGGAAGCTGGAACGAAGT	GCCAAACACTTGACCGTGAC
Mouse Upa	CTTCCCACTACCTTGGCTGG	AGACAGCAGTTCTCCCCAAG
Mouse Ccl2	AAAAACCTGGATCGGAACCAA	AGGTTTCTTCGCCACCTGAG
Mouse Mmp9	GTGTGTTCCCGTTCATCTTT	TTATCCTGGTCATAGTTGGCT
Mouse Fbxl6	TCTGGTTGGGGAGACCGTAT	TCTGGAGCTGAGAGAACCGA
Mouse Tkt	CATCATCGTGGACGGACACA	ATGTTTTTGGGGAGGGGCTT
Mouse Vrk2	GCCCAACAAAAAGAAAACTCAAGG	TGTACCGAGGCAGGGATCTT
Mouse Pd-l1	GGGCGTTTACTATCACGGCT	AGGGCAGCATTTCCCTTCAA
Mouse Gapdh	AGACGGCCGCATCTTCTT	TTCACACCGACCTTCACCAT
Human Icam1	CACGCCTCCCTGAACCTATC	ATCAGATGCGTGGCCTAGTG
Human Vcam1	AATGCCTGGGAAGATGGTCG	GATGTGGTCCCCTCATTCGT
Human Upa	CCAAAATGCTGTGTGCTGCT	GCCAGGCCATTCTCTTCCTT
Human Ccl2	CCCCAGTCACCTGCTGTTAT	GAGTTTGGGTTTGCTTGTCC
Human Mmp9	TTTGAGTCCGGTGGACGATG	GCTCCTCAAAGACCGAGTCC
Human Gapdh	TGGCACCGTCAAGGCTGAGAA	TGGTGAAGACGCCAGTGGACTC
